# Supplementary material for: Determinants of utilization of antenatal and delivery care at the community level in rural Bangladesh
Source: PLoS One. 2021 Sep 28;16(9):e0257782. doi: 10.1371/journal.pone.0257782 (PMC8478219; doi:10.1371/journal.pone.0257782)
Supplement: S1 Table — (DOCX) [file pone.0257782.s001.docx]

| **Characteristics (n=3293)** | **N (%)** |  | **Skilled Birth Attendance at delivery (n=3293)** | | | |
| --- | --- | --- | --- | --- | --- | --- |
|  |  | **n (%)** | **Unadjusted OR (95% CI)** | **P-value** | **Adjusted OR* (95% CI)** | **P-value** |
| **Age in years** | | | | | | |
| <20 | 581 (18) | 468 (81) | 1 |  | 1 |  |
| 20-30 | 2470 (75) | 1957 (79) | 0.92 (0.73 – 1.16) | 0.478 | 1.25 (0.93 - 1.66) | 0.133 |
| >30 | 242 (7) | 195 (81) | 1.00 (0.69 – 1.46) | 0.993 | 1.73 (1.10 - 2.74) | 0.019 |
| **Parity** | | | | | | |
| 0 | 1306 (40) | 1092 (84) | 1.79 (1.45 – 2.22) | <0.001 | 1.64 (1.25 - 2.16) | <0.001 |
| 1 | 1145 (35) | 905 (79) | 1.33 (1.07 – 1.63) | 0.008 | 1.25 (0.99 - 1.57) | 0.060 |
| ≥ 2 | 842 (26) | 623 (74) | 1 |  | 1 |  |
| **Years of education** | | | | | | |
| 0-5 | 426 (13) | 282 (66) | 1 |  | 1 |  |
| 6-10 | 2285 (69) | 1809 (79) | 1.94 (1.55 – 2.43) | <0.001 | 1.43 (1.13 - 1.83) | 0.004 |
| >10 | 582 (18) | 529 (91) | 5.10 (3.60 – 7.21) | <0.001 | 2.60 (1.76- 3.84) | <0.001 |
| **Husband education** | | | | | | |
| 0-5 | 888 (27) | 626 (71) | 1 |  | 1 |  |
| 6-10 | 1879 (57) | 1529 (81) | 1.83 (1.52 - 2.20) | <0.001 | 1.33 (1.09 - 1.63) | 0.006 |
| >10 | 526 (16) | 465 (88) | 3.19 (2.36 – 4.32) | <0.001 | 1.63 (1.16 - 2.28) | 0.005 |
| **Wealth index** | | | | | | |
| Poorest | 671 (20) | 462(69) | 1 |  | 1 |  |
| Poorer | 669 (20) | 498 (74) | 1.32 (1.04 - 1.67) | 0.023 | 1.16 (0.91 - 1.49) | 0.237 |
| Middle | 746 (23) | 619 (83) | 2.20 (1.71 – 2.83) | <0.001 | 1.82 (1.41 - 2.36) | <0.001 |
| Richer | 552 (17) | 463 (84) | 2.35 (1.78 – 3.11) | <0.001 | 1.81 (1.36 - 2.42) | <0.001 |
| Richest | 655 (20) | 578 (88) | 3.40 (2.54 – 4.53) | <0.001 | 2.34 (1.73 - 3.18) | <0.001 |

*Adjusted with women’s age, parity, education, husband’s education, and wealth index
