# Supplementary material for: Determinants of utilization of antenatal and delivery care at the community level in rural Bangladesh
Source: PLoS One. 2021 Sep 28;16(9):e0257782. doi: 10.1371/journal.pone.0257782 (PMC8478219; doi:10.1371/journal.pone.0257782)
Supplement: S1 File — (PDF) [file pone.0257782.s003.pdf]

eRegistry - Matlab Trial: Post-partum Home Visit Form

Date: |__|__| |__|__| |__|__|	Interviewers Code: |__|__|
DHIS2 ID #	

General information on participants in the eReg-Mat Trial

Time interview started: |__|__|:|__|__|			
Name of Woman:	Age: |__|__| Years		
Husband's name:	Age: |__|__| Years		
Address: Village: ______ Union: ______	Upazila:	1=Matlab North	2=Matlab South |__|
LMP: |__|__| |__|__| |__|__|	Mobile No:		
Pregnancy outcome date:	|__|__| |__|__| |__|__| DD MM YY		

No.	QUESTIONS & FILTERS	CODING RESPONSES			SKIP
**SECTION 1: MOST RECENT PREGNANCY INFORMATION**					
101	Pregnancy outcome	1=Alive	2=Stillbirth<br>3=Abortion/miscarriage/menstral regulation		201
102	Mode of delivery	Child 1	1= Normal delivery	2= C- section	|__|
		Child 2	1= Normal delivery	2= C- section	|__|
		Child 3	1= Normal delivery	2= C- section	|__|
103	What is the status of your child(ren) now?	Child 1	1= Alive	2= Dead	|__|
		Child 2	1= Alive	2= Dead	|__|
		Child 3	1= Alive	2= Dead	|__|
104	Date of neonatal death	Child 1	|__|__|-|__|__|-|__|__|		
		Child 2	|__|__|-|__|__|-|__|__|		
		Child 3	|__|__|-|__|__|-|__|__|		
105	Mother's current status	1= Alive		2= Dead	|__|
106	Date of maternal death	|__|__|-|__|__|-|__|__|			
**SECTION 2: PREVIOUS PREGNANCY INFORMATION**					
201	How many total pregnancies have you had, including live births, stillbirths, abortions, miscarriages, or those ended using menstrual regulation, including your most recent birth?	If none, record 00			|__|
202	How many total pregnancies have you had that ended in a live birth, including your most recent birth?	If none, record 00			|__|
203	How many total pregnancies have you had that ended in a stillbirth, including your most recent birth?	If none, record 00			|__|
204	How many living children do you have today, including your most recent birth?	If none, record 00			|__|
**SECTION 3: RECENT PREGNANCY**					
301	Now I would like to talk you about your <u>last</u> pregnancy. Did you receive check-up/antenatal care during this pregnancy?	1=Yes			|__|
		2=No			501
		9=Don't Remember			501
302	How many times did you seek antenatal care during your pregnancy?	|__|__| times			
		99= Unknown			
303	How long does it normally take to get from home to antenatal care?	|__|__| hours			
		|__|__| minutes			

304	Do you have your ANC card?	1=Yes — If yes, enter all dates below, and probe for more<br>2=No — If no, use a calendar to estimate dates to enter below	|__|
305	When was your first ANC visit?	Date: |__|__| |__|__| |__|__|	
306	Whom did you see?	1= Doctors<br>2= Nurse/Midwife<br>3= Family Welfare Assistant<br>4= Family Welfare Visitor<br>5= Traditional Birth Attendant<br>6= Community Health Care Provider<br>7= Health Assistant<br>8= Village Doctor<br>9= SACMO<br>10= Others (Specify)	|__|
307	Where did you have that ANC visit?<br>Facility:________________	1=Home<br>2=H & FWC<br>3=Community Clinic<br>4=UHC<br>5=Other public facility<br>6=Other private facility<br>7= Satellite Clinic<br>8=Other (specify)	|__|
308	When was your second ANC visit?	Date: |__|__| |__|__| |__|__|	
309	Whom did you see?	1= Doctors<br>2= Nurse/Midwife<br>3= Family Welfare Assistant<br>4= Family Welfare Visitor<br>5= Traditional Birth Attendant<br>6= Community Health Care Provider<br>7= Health Assistant<br>8= Village Doctor<br>9= SACMO<br>10= Others (Specify)	|__|
310	Where did you have your ANC visit?<br>Facility:________________	1=Home<br>2=H & FWC<br>3=Community Clinic<br>4=UHC<br>5=Other public facility<br>6=Other private facility<br>7= Satellite Clinic<br>8=Other (specify)	|__|
311	When was your third ANC visit?	Date: |__|__| |__|__| |__|__|	
312	Whom did you see?	1= Doctors<br>2= Nurse/Midwife<br>3= Family Welfare Assistant<br>4= Family Welfare Visitor<br>5= Traditional Birth Attendant<br>6= Community Health Care Provider<br>7= Health Assistant<br>8= Village Doctor<br>9= SACMO<br>10= Others (Specify)	|__|

313	Where did you have your ANC visit?<br>Facility:____________________	1=Home<br>2=H & FWC<br>3=Community Clinic<br>4=UHC<br>5=Other public facility<br>6=Other private facility<br>7= Satellite Clinic<br>8=Other (specify)	|__|
314	When was your fourth ANC visit?	Date: |__|__||__|__||__|__|	
315	Whom did you see?	1= Doctors<br>2= Nurse/Midwife<br>3= Family Welfare Assistant<br>4= Family Welfare Visitor<br>5= Traditional Birth Attendant<br>6= Community Health Care Provider<br>7= Health Assistant<br>8= Village Doctor<br>9= SACMO<br>10= Others (Specify)	|__|
316	Where did you have your ANC visit?<br>Facility:____________________	1=Home<br>2=H & FWC<br>3=Community Clinic<br>4=UHC<br>5=Other public facility<br>6=Other private facility<br>7= Satellite Clinic<br>8=Other (specify)	|__|
317	When was your fifth ANC visit?	Date: |__|__||__|__||__|__|	
318	Whom did you see?	1= Doctors<br>2= Nurse/Midwife<br>3= Family Welfare Assistant<br>4= Family Welfare Visitor<br>5= Traditional Birth Attendant<br>6= Community Health Care Provider<br>7= Health Assistant<br>8= Village Doctor<br>9= SACMO<br>10= Others (Specify)	|__|
319	Where did you have your ANC visit?<br>Facility:____________________	1=Home<br>2=H & FWC<br>3=Community Clinic<br>4=UHC<br>5=Other public facility<br>6=Other private facility<br>7= Satellite Clinic<br>8=Other (specify)	|__|
320	When was your sixth ANC visit?	Date: |__|__||__|__||__|__|	
321	Whom did you see?	1= Doctors<br>2= Nurse/Midwife<br>3= Family Welfare Assistant<br>4= Family Welfare Visitor	|__|

		5= Traditional Birth Attendant	
		6= Community Health Care Provider	
		7= Health Assistant	
		8= Village Doctor	
		9= SACMO	
		10= Others (Specify)	
322	Where did you have your ANC visit? Facility:______________	1=Home	|__|
		2=H & FWC	
		3=Community Clinic	
		4=UHC	
		5=Other public facility	
		6=Other private facility	
		7= Satellite Clinic	
		8=Other (specify)	

If there are more than 6 ANC visits, complete an additional sheet of paper.

SECTION 5: DELIVERY

Now I would like to ask you some questions about the delivery of (NAME).

501	Where did you give birth to (NAME)?	1=Home	|__|
		2=H & FWC	
		3=Community Clinic	
		4=UHC	
		5=Other public facility	
		6=Other private facility	
		7=Other (specify)	
502	Who delivered the baby?	1= Doctors	|__|
		2= Nurse/Midwife	
		3= Family Welfare Assistant	
		4= Family Welfare Visitor	
		5= Traditional Birth Attendant	
		6= Community Health Care Provider	
		7= Health Assistant	
		8= Village Doctor	
		9= SACMO	
		10= Others (Specify)	

SECTION 9: SOCIO-ECOMIC STATUS

901	Women's education	**Types of institution**	**Years of education**
		1= School- Formal	|__|__|
		2= School-Nonformal	|__|__|
		3= Madrassah-Registered	|__|__|
		4= Madrassah-Unregistered	|__|__|
902	Husband's education	**Types of institution**	**Years of education**
		1= School- Formal	|__|__|
		2= School-Nonformal	|__|__|
		3= Madrassah-Registered	|__|__|
		4= Madrassah-Unregistered	|__|__|

Information on Land:

903	How much land do your household own?	**Decimal**	**Kani**	**Gonda**	**Kora**
	Homestead (including ponds & ditches)	|__|__|__|__|__|	|__|__|__|__|__|	|__|__|__|__|__|	|__|__|__|__|__|
	Agricultural land	|__|__|__|__|__|	|__|__|__|__|__|	|__|__|__|__|__|	|__|__|__|__|__|
			1 Kani= Decimal	1 Gonda= Decimal	1 Kora= Decimal

Source of Income:

904	a. During the last 12 months what were the sources of income of your household?	1. Agriculture (Own land)	
		2. Agriculture (Share crops)	
		3. Land/ pond mortage/ lease/ rent (in/out)	
		4. Day labourer/ Rickshaw/ Van puller	
		5. Skilled labourer (driver, carpenter,plumber, electrician)	
		6. Selling/Catching Fish /Fish Farming	
		7. Cattle/ Chicken/ Duck/ Poultry/Selling Milk	
		8. Selling Handicrafts	
	RECORD ALL RESPONSES	9. Tailoring Work	
		10. Business (Small)	
		11. Business (MEDIUM)	
		12. Various Business (BIG)	
		13. Paid Employment	
	b. What was the source of income?	14. Pension	
		15. Remittance (within country)	
	i.	16. Remittance (Outside country)	
	ii.	17. Food for work	
	iii.	18. Old age/ destitute (allowances)/ VGD/ Other allowances (Freedom fighter), Chairman, Member	
	vi.		
	v.	19. House/ Shop rent	
		20. Rent of Taxi, Tempo, Auto	
		21. Boat/ Rickshaw/ van renting out	
		22. Interest Business	
		23. Journalist	
		24. Others (specify)	

Food security:

905	Some households may not have food for all members three times every day for all times). Has your household been able to have food for all members for three times every day during the last one year?	1=Yes	2=No	

Housing Facility:

906	a. How many rooms does your household have?		
	b. Materials used for construction of the main dwelling	**Items**	
		1= Hemp/hay/bamboo, or other	
		2= Mud brick, or C.I. sheet/wood	
	(Write by observing)	3= Brick/cement	

Commodities/ Assets

907	a. Does your household own the following commodities or assets?	Rickshaw/ Rickshaw van	1=Yes	2=No	
		Auto rickshaw (CNG/ Battery/ Patrol)	1=Yes	2=No	
		Power tiller (Cultivation)	1=Yes	2=No	
		Pump (irrigation / water pump)	1=Yes	2=No	
		Computer	1=Yes	2=No	
		Solar panel	1=Yes	2=No	
	RECORD ALL RESPONSES	Almirah/ Showcase	1=Yes	2=No	
		Sofa set	1=Yes	2=No	
		Television	1=Yes	2=No	
		Mobile phone	1=Yes	2=No	
		Bi-cycle	1=Yes	2=No	
		Motor cycle	1=Yes	2=No	
		Refrigerator	1=Yes	2=No	
		Fan	1=Yes	2=No	

	b. How many fans does your household own?				|__|__|	
	c. How many mobile phones does your household own?				|__|__|	
	d. Do you own/use your own phone?		1=Yes	2=No		|__|
**Livestock**						
908	Does your household own livestocks?		1=Yes	2=No		|__|
909	If yes, please mention type and number?		**Type**		**Number**	
		1	Duck and Chicken		|__|__|	
		2	Goat and Sheep		|__|__|	
		3	Cow and Buffalo		|__|__|	
**Latrine**						
910	Do you have any latrine?		1=Yes	2=No		|__|

911	What types of latrines are used by your household?<br><br>(Write by observing)	1= Septic tank/ modern toilet<br>2= Ring/Slab but waste not drained out<br>3= Ring/Slab but waste drained out<br>4= Concrete latrine but waste drained out<br>5= Kancha (Earth) latrine (open)<br>6= Open/Bush<br>7= Others (Specify)	
**Drinking Water (with in 1 year)**			
912	What are the main source (most commonly used) of drinking water?	1= Deep Tubewell<br>2= Tubewell (Green)<br>3= Tubewell (red)<br>4= Tubewell not tested yet<br>5= Supply water<br>6= Rain water<br>7= Pond<br>8= River<br>9= Ditch/ canal<br>10= Others (specify)	|__|
**Source of Light**			
913	Main source of power/ electricity?	1= Kerosine Oil<br>2= Electricity<br>3= Solar Panel<br>4= Generator<br>5= Others (specify)	|__|
**Fuel**			
914	Type of fuel mainly used for cooking	1= Wood/Wood dust/Paddy Husk<br>2= Leaves and straw<br>3= Kerosine (stove)<br>4= Gas (line)<br>5=Gas (Cylinder)<br>6= Biogas<br>7= Cowdung<br>8= Electric (Oven/ rice cooker/ micro-oven/ grill)<br>9= Others	|__|

End time: |__|__|:|__|__|

Edited By: **Code:** |__|__| **Entry By:** **Code:** |__|__|

Date: |__|__| |__|__| |__|__| **Date:** |__|__| |__|__| |__|__|

icddr,b

eRegistry - Matlab Trial: Post-partum Home Visit Form

তারিখ: |___|___|-|___|___|-|___|___| সাক্ষাৎকারগ্রহণকারীর কোড: |___|___|

DHIS2 ID#

eReg-Mat ট্রায়াল এ অংশগ্রহণকারীদের সাধারণ তথ্য

সাক্ষাৎকার শুরুর সময় ঃ |___|___|:|___|___|					
মায়ের নাম:			মায়ের বয়স: |___|___| বছর		
স্বামীর নামঃ			স্বামীর বয়স: |___|___| বছর		
ঠিকানাঃ	গ্রাম- ______________	ইউনিয়ন- __________	উপজেলাঃ	1= মতলব উত্তর 2= মতলব দক্ষিন	|___|
গর্ভের ফলাফলের তারিখ ঃ |___|___|-|___|___|-|___|___| দিন মাস বছর					

No.	QUESTIONS & FILTERS	CODING RESPONSES			SKIP
**Section 1: Most recent pregnancy information (সাম্প্রতিক গর্ভাবস্থার তথ্য)**					
101	গর্ভের ফলাফল	1= জীবিত ↓	2=মৃত প্রসব<br>3=গর্ভপাত/ মাসিক নিয়মিতকরণ (MR)	→ 201	|___|
102	প্রসবের ধরণঃ	বাচ্চা-১	1= নরমাল ডেলিভারী	2= সিজারিয়ান সেকশন	|___|
		বাচ্চা-২	1= নরমাল ডেলিভারী	2= সিজারিয়ান সেকশন	|___|
		বাচ্চা-৩	1= নরমাল ডেলিভারী	2= সিজারিয়ান সেকশন	|___|
103	বাচ্চাটি এখন কি অবস্থায় আছে ?	বাচ্চা-১	1= জীবিত	2= মৃত	|___|
		বাচ্চা-২	1= জীবিত	2= মৃত	|___|
		বাচ্চা-৩	1= জীবিত	2= মৃত	|___|
104	নবজাতকের মৃত্যুর তারিখ	বাচ্চা-১	|___|___|-|___|___|-|___|___|		
		বাচ্চা-২	|___|___|-|___|___|-|___|___|		
		বাচ্চা-৩	|___|___|-|___|___|-|___|___|		
105	মায়ের বর্তমান অবস্থা	1= জীবিত		2= মৃত	|___|
106	মায়ের মৃত্যুর তারিখ: |___|___|-|___|___|-|___|___|				
**Section 2: Previous Pregnancy Information (পূর্বের গর্ভের তথ্য)**					
201	বর্তমান প্রসবসহ জীবিত বাচ্চা প্রসব, মৃত বাচ্চা প্রসব, গর্ভপাত অথবা মাসিক নিয়মিতকরণের মাধ্যমে মোট কতবার আপনি গর্ভধারণ করেছিলেন?				|___|
202	বর্তমান প্রসবসহ মোট কতবার আপনি জীবিত বাচ্চা প্রসব করেছিলেন?				|___|
203	বর্তমান প্রসবসহ মোট কতবার আপনি মৃত বাচ্চা প্রসব করেছিলেন?				|___|
204	সাম্প্রতিক বাচ্চাসহ বর্তমানে কতজন বাচ্চা জীবিত আছে?	যদি প্রযোজ্য না হয়, তাহলে ‘00’ রেকর্ড করুন			|___|

Section 3: Recent Pregnancy (সর্বশেষ গর্ভাবস্থা)

301	এখন আমি আপনার সঙ্গে শেষ গর্ভাবস্থা নিয়ে কথা বলতে চাই। আপনি কি সর্বশেষ গর্ভের সময় গর্ভকালীন সেবা (ANC) নিয়েছিলেন?	1= হ্যাঁ<br>2= না → 501<br>9= মনে নাই → 501	|___|
302	গর্ভাবস্থায় আপনি কতবার গর্ভকালীন সেবা নিয়েছিলেন?	|___|___| বার<br>99 = জানা নেই	
303	গর্ভকালীন সেবা গ্রহনের জন্য আপনার বাসা থেকে স্বাস্থ্যকেন্দ্রে যেতে সাধারণত কতক্ষণ সময় প্রয়োজন	সময়ঃ |___|___| ঘন্টা<br>|___|___| মিনিট	
304	আপনার কি ANC কার্ড আছে?	1= হ্যাঁ, যদি হ্যাঁ হয়, তাহলে কার্ড দেখে সকল তারিখ লিখুন এবং আরো আছে কি না তা অনুসন্ধান করুন<br>2= না, যদি না হয়, তাহলে নিচে সম্ভাব্য তারিখ বসানোর জন্য একটি ক্যালেন্ডার ব্যবহার করুন	|___|
305	আপনি প্রথম গর্ভকালীন সেবা (ANC) কখন নিয়েছিলেন?	তারিখঃ |___|___|-|___|___|-|___|___|	
306	আপনি প্রথম গর্ভকালীন সেবা কার কাছ থেকে নিয়েছিলেন ?	1= Doctors<br>2 = Nurse/Midwife<br>3 = Family Welfare Assistant<br>4 = Family Welfare Visitor<br>5 = Traditional Birth Attendant<br>6 = Community Health Care Provider<br>7 = Health Assistant<br>8 = Village Doctor<br>9 = SACMO<br>10 = Others (Specify) ______________________	|___|
307	আপনি কোথায় প্রথম গর্ভকালীন সেবা নিয়েছিলেন?<br><br>স্বাস্থ্যকেন্দ্রের নাম:______________________	1= বাড়িতে<br>2= স্বাস্থ্য ও পরিবার কল্যাণ কেন্দ্র<br>3= কমিউনিটি ক্লিনিক<br>4= উপজেলা স্বাস্থ্যকেন্দ্র<br>5= অন্যান্য সরকারী স্বাস্থ্যকেন্দ্র<br>6= অন্যান্য বেসরকারী স্বাস্থ্যকেন্দ্র<br>7= স্যাটেলাইট ক্লিনিক<br>8= অন্যান্য (উল্লেখ করুন)______________________	|___|
308	আপনি দ্বিতীয় গর্ভকালীন সেবা (ANC) কখন নিয়েছিলেন?	তারিখঃ |___|___|-|___|___|-|___|___|	

<table>
<tr><td rowspan="10">309</td><td rowspan="10">আপনি দ্বিতীয় গর্ভকালীন সেবা কার কাছ থেকে নিয়েছিলেন?</td><td>1 = Doctors</td><td rowspan="10">|___|</td></tr>
<tr><td>2 = Nurse/Midwife</td></tr>
<tr><td>3 = Family Welfare Assistant</td></tr>
<tr><td>4 = Family Welfare Visitor</td></tr>
<tr><td>5 = Traditional Birth Attendant</td></tr>
<tr><td>6 = Community Health Care Provider</td></tr>
<tr><td>7 = Health Assistant</td></tr>
<tr><td>8 = Village Doctor</td></tr>
<tr><td>9 = SACMO</td></tr>
<tr><td>10 = Others (Specify) ______________________________</td></tr>
<tr><td rowspan="8">310</td><td rowspan="8">আপনি কোথায় দ্বিতীয় গর্ভকালীন সেবা নিয়েছিলেন?

স্বাস্থ্যকেন্দ্রের নাম:____________________________</td><td>1= বাড়িতে</td><td rowspan="8">|___|</td></tr>
<tr><td>2= স্বাস্থ্য ও পরিবার কল্যাণ কেন্দ্র</td></tr>
<tr><td>3= কমিউনিটি ক্লিনিক</td></tr>
<tr><td>4= উপজেলা স্বাস্থ্যকেন্দ্র</td></tr>
<tr><td>5= অন্যান্য সরকারী স্বাস্থ্যকেন্দ্র</td></tr>
<tr><td>6= অন্যান্য বেসরকারী স্বাস্থ্যকেন্দ্র</td></tr>
<tr><td>7= স্যাটেলাইট ক্লিনিক</td></tr>
<tr><td>8= অন্যান্য (উল্লেখ করুন)______________________________</td></tr>
<tr><td>311</td><td>আপনি তৃতীয় গর্ভকালীন সেবা (ANC) কখন নিয়েছিলেন?</td><td colspan="2">তারিখ: |___|___|-|___|___|-|___|___|</td></tr>
<tr><td rowspan="10">312</td><td rowspan="10">আপনি তৃতীয় গর্ভকালীন সেবা কার কাছ থেকে নিয়েছিলেন?</td><td>1 = Doctors</td><td rowspan="10">|___|</td></tr>
<tr><td>2 = Nurse/Midwife</td></tr>
<tr><td>3 = Family Welfare Assistant</td></tr>
<tr><td>4 = Family Welfare Visitor</td></tr>
<tr><td>5 = Traditional Birth Attendant</td></tr>
<tr><td>6 = Community Health Care Provider</td></tr>
<tr><td>7 = Health Assistant</td></tr>
<tr><td>8 = Village Doctor</td></tr>
<tr><td>9 = SACMO</td></tr>
<tr><td>10 = Others (Specify) ______________________________</td></tr>
<tr><td rowspan="4">313</td><td rowspan="4">আপনি কোথায় তৃতীয় গর্ভকালীন সেবা নিয়েছিলেন?

স্বাস্থ্যকেন্দ্রের নাম:____________________________</td><td>1= বাড়িতে</td><td rowspan="4">|___|</td></tr>
<tr><td>2= স্বাস্থ্য ও পরিবার কল্যাণ কেন্দ্র</td></tr>
<tr><td>3= কমিউনিটি ক্লিনিক</td></tr>
<tr><td>4= উপজেলা স্বাস্থ্যকেন্দ্র</td></tr>
</table>

		5= অন্যান্য সরকারী স্বাস্থ্যকেন্দ্র	
		6= অন্যান্য বেসরকারী স্বাস্থ্যকেন্দ্র	
		7= স্যাটেলাইট ক্লিনিক	
		8= অন্যান্য (উল্লেখ করুন)________________________	
314	আপনি চতুর্থ গর্ভকালীন সেবা (ANC) কখন নিয়েছিলেন?	তারিখ: |___|___|-|___|___|-|___|___|	
315	আপনি চতুর্থ গর্ভকালীন সেবা কার কাছ থেকে নিয়েছিলেন?	1 = Doctors	|___|
		2 = Nurse/Midwife	
		3 = Family Welfare Assistant	
		4 = Family Welfare Visitor	
		5 = Traditional Birth Attendant	
		6 = Community Health Care Provider	
		7 = Health Assistant	
		8 = Village Doctor	
		9 = SACMO	
		10 = Others (Specify) ____________________________	
316	আপনি কোথায় চতুর্থ গর্ভকালীন সেবা নিয়েছিলেন?	1= বাড়িতে	|___|
		2= স্বাস্থ্য ও পরিবার কল্যাণ কেন্দ্র	
		3= কমিউনিটি ক্লিনিক	
	স্বাস্থ্যকেন্দ্রের নাম:______________________	4= উপজেলা স্বাস্থ্যকেন্দ্র	
		5= অন্যান্য সরকারী স্বাস্থ্যকেন্দ্র	
		6= অন্যান্য বেসরকারী স্বাস্থ্যকেন্দ্র	
		7= স্যাটেলাইট ক্লিনিক	
		8= অন্যান্য (উল্লেখ করুন)________________________	
317	আপনি পঞ্চম গর্ভকালীন সেবা (ANC) কখন নিয়েছিলেন?	তারিখ: |___|___|-|___|___|-|___|___|	
318	আপনি পঞ্চম গর্ভকালীন সেবা কার কাছ থেকে নিয়েছিলেন?	1 = Doctors	|___|
		2 = Nurse/Midwife	
		3 = Family Welfare Assistant	
		4 = Family Welfare Visitor	
		5 = Traditional Birth Attendant	
		6 = Community Health Care Provider	
		7 = Health Assistant	
		8 = Village Doctor	

		9 = SACMO	
		10 = Others (Specify) ________________	
319	আপনি কোথায় পঞ্চম গর্ভকালীন সেবা নিয়েছিলেন?<br><br>স্বাস্থ্যকেন্দ্রের নাম:________________	1= বাড়িতে<br>2= স্বাস্থ্য ও পরিবার কল্যাণ কেন্দ্র<br>3= কমিউনিটি ক্লিনিক<br>4= উপজেলা স্বাস্থ্যকেন্দ্র<br>5= অন্যান্য সরকারী স্বাস্থ্যকেন্দ্র<br>6= অন্যান্য বেসরকারী স্বাস্থ্যকেন্দ্র<br>7= স্যাটেলাইট ক্লিনিক<br>8= অন্যান্য (উল্লেখ করুন)________________	|___|
320	আপনি ষষ্ঠ গর্ভকালীন সেবা (ANC) কখন নিয়েছিলেন?	তারিখঃ |___|___|-|___|___|-|___|___|	
321	আপনি ষষ্ঠ গর্ভকালীন সেবা কার কাছ থেকে নিয়েছিলেন?	1= Doctors<br>2= Nurse/Midwife<br>3= Family Welfare Assistant<br>4= Family Welfare Visitor<br>5= Traditional Birth Attendant<br>6= Community Health Care Provider<br>7= Health Assistant<br>8= Village Doctor<br>9= SACMO<br>10 = Others (Specify) ________________	|___|
322	আপনি কোথায় ষষ্ঠ গর্ভকালীন সেবা নিয়েছিলেন?<br><br>স্বাস্থ্যকেন্দ্রের নাম:________________	1= বাড়িতে<br>2= স্বাস্থ্য ও পরিবার কল্যাণ কেন্দ্র<br>3= কমিউনিটি ক্লিনিক<br>4= উপজেলা স্বাস্থ্যকেন্দ্র<br>5= অন্যান্য সরকারী স্বাস্থ্যকেন্দ্র<br>6= অন্যান্য বেসরকারী স্বাস্থ্যকেন্দ্র<br>7= স্যাটেলাইট ক্লিনিক<br>8= অন্যান্য (উল্লেখ করুন)________________	|___|
**ছয়টির বেশি গর্ভকালীন সেবার ক্ষেত্রে, তথ্যসমূহ আলাদা পৃষ্ঠায় পূরণ করুন।**			
**Section 5: Delivery (প্রসব সেবা)**			
**এখন আমি আপনাকে (নাম) ডেলিভারি সম্পর্কে কিছু প্রশ্ন জিজ্ঞাসা করতে চাই।**			
501	আপনি কোথায় প্রসব করেছিলেন?	1= বাড়িতে<br>2= স্বাস্থ্য ও পরিবার কল্যাণ কেন্দ্র<br>3= কমিউনিটি ক্লিনিক<br>4= উপজেলা স্বাস্থ্যকেন্দ্র	|___|

		5= অন্যান্য সরকারী স্বাস্থ্যকেন্দ্র	
		6= অন্যান্য বেসরকারী স্বাস্থ্যকেন্দ্র	
		7= অন্যান্য (উল্লেখ করুন) ______________________________	
502	প্রসব কে করিয়েছিলেন ?	1 = Doctors	|___|
		2 = Nurse/Midwife	
		3 = Family Welfare Assistant	
		4 = Family Welfare Visitor	
		5 = Traditional Birth Attendant	
		6 = Community Health Care Provider	
		7 = Health Assistant	
		8 = Village Doctor	
		9 = SACMO	
		10 = Others (Specify) _________________________	

Section 9: Socio-economic status (আর্থ-সামাজিক অবস্থা)

		শিক্ষা প্রতিষ্ঠানের ধরণ	কত বছর পড়াশুনা করেছেন
901	মায়ের শিক্ষা ঃ	1= আনুষ্ঠানিক স্কুল	|___|___|
		2= উপনুষ্ঠানিক স্কুল	|___|___|
		3= নিবন্ধিত মাদ্রাসা	|___|___|
		4= অনিবন্ধিত মাদ্রাসা	|___|___|
902	স্বামীর শিক্ষা ঃ	**শিক্ষা প্রতিষ্ঠানের ধরণ**	**কত বছর পড়াশুনা করেছেন**
		1= আনুষ্ঠানিক স্কুল	|___|___|
		2= উপনুষ্ঠানিক স্কুল	|___|___|
		3= নিবন্ধিত মাদ্রাসা	|___|___|
		4= অনিবন্ধিত মাদ্রাসা	|___|___|

ভূমি সম্পর্কিত তথ্য:

		ডেসিমাল/শতক	কানি	গন্ডা	কড়া
903	আপনার খানায় জমির পরিমান কত?				
	বসত জমি (পুকুর/ডোবা সহ)	|__|__|__|.|__|__|	|__|__|__|.|__|__|	|__|__|__|.|__|__|	|__|__|__|.|__|__
	কৃষি জমি	|__|__|__|.|__|__|	|__|__|__|.|__|__|	|__|__|__|.|__|__|	|__|__|__|.|__|__
			১ কানি = শতক	১ গন্ডা = শতক	১ কড়া = শতক

আয়ের উৎস:

904	a. গত ১২ মাসে আপনার খানায় আয়ের উৎস কি কি ছিল?	1. কৃষি (নিজের জমি)	
		2. কৃষি (বর্গা ফসল)	
		3. জমি/পুকুর গচ্ছিত/চুক্তিকৃত/ভাড়া দেয়া/নেয়া	
		4. দিনমজুর/রিক্সা চালক/ভ্যান চালক	
		5. দক্ষ শ্রমিক (ড্রাইভার/কাঠমিস্ত্রি/স্যানিটারী মিস্ত্রি/ইলেকট্রিক মিস্ত্রি)	
		6. মাছ ধরে বিক্রি করে/মাছ চাষ	
		7. গবাদি পশু/হাস-মুরগী/পোল্ট্রি খামার/দুধ বিক্রি	

		8. কুটির শিল্প/হস্ত শিল্প	
	সকল উত্তর রেকর্ড করুন	9. দর্জি	
		10. ক্ষুদ্র ব্যবসা	
		11. মাঝারী ব্যবসা	
		12. ব্যবসা (বিভিন্ন ধরনের বড় ব্যবসা)	
		13. চাকুরী	
	b. আয়ের প্রধান উৎস কি	14. পেনশন	
	ছিল?	15. দেশের মধ্যে থেকে টাকা	
	i. |___|___|	16. বিদেশ থেকে টাকা	
	ii. |___|___|	17. কাজের বিনিময়ে খাদ্য	
	iii. |___|___|	18. বয়স্ক ভাতা/দুস্থ ভাতা/ভিজিডি ভাতা/মুক্তিযোদ্ধা ভাতা/চেয়ারম্যান/মেম্বার	
	iv. |___|___|	19. বাড়ি/দোকান ভাড়া	
	v. |___|___|	20. ট্যাক্সি/টেম্পো/অটো ভাড়া থেকে আয়	
		21. নৌকা/রিক্সা/ভ্যান ভাড়া থেকে আয়	
		22. সুদের ব্যবসা	
		23. সাংবাদিক	
		24. অন্যান্য (উল্লেখ করুন) ____________________________	

খাদ্য নিরাপত্তাঃ

905	(কিছু পরিবার সব সময় তিনবার প্রত্যেক সদস্যদের জন্য খাবার ব্যবস্থা করতে পারে না।) গত ১ বছরে আপনার পরিবারের সব সদস্যের জন্য দৈনিক তিনবেলা খাবার ব্যবস্থা করতে সক্ষম হয়েছিলেন?	1= হ্যাঁ	2= না	|___|

বাসস্থানঃ

906	a. আপনার বাসায়/ঘরে কতটি কক্ষ আছে?		|___|___|
	b. প্রধান কক্ষের দেয়াল নির্মাণে ব্যবহৃত প্রধান উপাদান কি?	উপাদান সমূহ	
		1= ছন/খড়/বাঁশ, বা অন্যান্য	|___|
		2= মাটির তৈরি ইট, অথবা টিন/ কাঠ	|___|
	(পর্যবেক্ষন করে লিখুন)	3= ইট/সিমেন্ট	|___|

পণ্য/সম্পদ

907	ক) আপনার পরিবারের মালিকানায় নিম্নলিখিত কি কি পণ্য বা সম্পদ রয়েছে?	রিক্সা / রিক্সা ভ্যান	1= হ্যাঁ	2= না	|___|
		অটো রিক্সা (সিএনজি / ব্যাটারী / পেট্রোল)	1= হ্যাঁ	2= না	|___|
		পাওয়ার টিলার (চাষের)	1= হ্যাঁ	2= না	|___|
		পাম্প সেচ/ওয়াটার পাম্প	1= হ্যাঁ	2= না	|___|
		কম্পিউটার	1= হ্যাঁ	2= না	|___|
		সৌর প্যানেল	1= হ্যাঁ	2= না	|___|
	সকল উত্তর রেকর্ড করুন	আলমারী / শোকেস	1= হ্যাঁ	2= না	|___|
		সোফা সেট	1= হ্যাঁ	2= না	|___|
		টিভি	1= হ্যাঁ	2= না	|___|
		মোবাইল ফোন	1= হ্যাঁ	2= না	|___|
		বাই-সাইকেল	1= হ্যাঁ	2= না	|___|
		মোটর সাইকেল	1= হ্যাঁ	2= না	|___|

		ফ্রিজ	1= হ্যাঁ	2= না	|___|
		ফ্যান	1= হ্যাঁ	2= না	|___|
	খ) আপনার বাড়িতে কয়টি ফ্যান আছে?			|___|___|	
	গ) আপনার বাড়িতে কয়টি মোবাইল ফোন আছে?			|___|___|	
	ঘ) আপনার কি নিজস্ব মোবাইল ফোন আছে/ আপনার নিজস্ব ফোন কি আপনি নিজে ব্যবহার করেন?		1= হ্যাঁ	2= না	|___|
**গবাদি পশুপালন:**					
908	আপনার খানায় কোন গবাদি পশু আছে?		1= হ্যাঁ	2= না	|___|
909	যদি হ্যাঁ হয়, তাহলে এর ধরণ এবং নম্বর উল্লেখ করুন?		ধরন	সংখ্যা	
			1= হাঁস বা মুরগী	|___|___|	
			2= ছাগল বা ভেড়া	|___|___|	
			3= গরু বা মহিষ	|___|___|	
**পায়খানা**					
910	আপনাদের কি কোন পায়খানা / ল্যাট্রিন আছে?		1= হ্যাঁ	2= না	|___|
911	আপনার বাড়িতে কি ধরনের পায়খানা / ল্যাট্রিন ব্যবহার করা হয়?	1= সেপ্টিক ট্যাংক / আধুনিক টয়লেট			|___|
		2= রিং/ স্লাব কিন্তু বর্জ্য পাইপের মাধ্যমে নিষ্কাশন হয় না			|___|
		3= রিং / স্লাব কিন্তু বর্জ্য পাইপের মাধ্যমে নিষ্কাশন হয়			|___|
		4= পাকা পায়খানা (কংক্রিট ল্যাট্রিন), কিন্তু বর্জ্য পাইপের মাধ্যমে নিষ্কাশন হয়			|___|
	(পর্যবেক্ষন করে লিখুন)	5= খোলা মাটির পায়খানা (কাচা পায়খানা)			|___|
		6= খোলা জায়গায়/ ঝোপঝোড়ে			|___|
		7= অন্যান্য (উল্লেখ করুন) ________________________			|___|
**পানীয় জলের উৎস: (১ বছরের মধ্যে)**					
912	আপনাদের খাবার পানির প্রধান উৎস কি?	1= গভীর নলকুপ (Deep Tubewell)			|___|
		2= টিউবওয়েল (সবুজ)			
		3= টিউবওয়েল (লাল)			
		4= টিউবওয়েল (এখনো পরীক্ষা করা হয় নি)			
		5= সাপ্লাই পানি			
		6= বৃষ্টির পানি			
		7= পুকুর			
		8= নদী			
		9= খনন / খাল			
		10= অন্যান্য (উল্লেখ করুন) ________________________			
**আলোর উৎস**					
913	আপনার বাড়িতে আলোর প্রধান উৎস কি?	1= কেরোসিন তেল			|___|
		2= বিদ্যুৎ			
		3= সৌর প্যানেল			
		4= জেনারেটর			
		5= অন্যান্য (উল্লেখ করুন) ________________________			

জ্বালানি			
914	রান্না করার জন্য ব্যবহৃত জ্বালানীর ধরন	1= কাঠ/কাঠের টুকরো/ধানের তুস	|___|
		2= পাতা এবং খড়	|___|
		3= কেরোসিন (চুলা)	|___|
	ব্যবহৃত জ্বালানী ক্রম অনুযায়ী লিখুনঃ	4= গ্যাস (লাইন)	|___|
		5= গ্যাস (সিলিন্ডার)	|___|
		6= বায়োগ্যাস	|___|
		7= গোবর	|___|
		8= বৈদুতিক (ওভেন/রাইস কুকার/মাইক্রো-ওভেন/গ্রিল)	|___|
		9= অন্যান্য ____	

সাক্ষাৎকার শেষ সময়: |__|__|:|__|__| **সাক্ষাৎকার গ্রহণকারীর নামঃ**

Edited By: **Code: |___|___|** **Entry By:** **Code: |___|___|**

Date |___|___|-|___|___|-|___|___| Date: |___|___|-|___|___|-|___|___|
