# Supplementary material for: Determinants of utilization of antenatal and delivery care at the community level in rural Bangladesh
Source: PLoS One. 2021 Sep 28;16(9):e0257782. doi: 10.1371/journal.pone.0257782 (PMC8478219; doi:10.1371/journal.pone.0257782)
Supplement: Attachment — Submitted filename: PONE-D-21-17032_reviewer.pdf [file pone.0257782.s004.pdf]

PLOS ONE

Determinants of utilization of antenatal and delivery care at the community level in rural Bangladesh

--Manuscript Draft--

**Manuscript Number:**	PONE-D-21-17032
**Article Type:**	Research Article
**Full Title:**	Determinants of utilization of antenatal and delivery care at the community level in rural Bangladesh
**Short Title:**	Determinants of utilization of maternal care at the community level in rural Bangladesh
**Corresponding Author:**	Jesmin Pervin<br>International Centre for Diarrhoeal Disease Research Bangladesh<br>Dhaka, BANGLADESH
**Keywords:**	Antenatal Care, Facility Delivery, Timely first ANC, Timely four ANC visits, Cross-sectional study
**Abstract:**	Background: Timely utilization of antenatal care and delivery services supports the health of mothers and babies. This study aims to assess the utilization, timeliness of, and socio-demographic determinants of antenatal and delivery care services in two sub-districts in Bangladesh.<br>Methods: This cross-sectional study used data collected through a structured questionnaire in the eRegMat cluster-randomized controlled trial, which enrolled pregnant women between October 2018-June 2020. Bivariate and multivariate logistic regression were employed. To determine the associations of socio-demographic variables with timely first ANC, four timely ANC visits, and facility delivery, binary logistic regression was executed.<br>Results: Data were available on 3293 pregnant women. Attendance at a timely first antenatal care visit was 59%. Uptake of four timely antenatal care visits was 4.2%. About three-fourths of the women delivered at a health facility. Women from all socioeconomic groups gradually shifted to utilizing private hospitals from the public health facilities, as the pregnancy advanced. Timely first antenatal care visit was associated with: women over 30 years of age (AOR: 1.52, 95% CI: 1.05 - 2.20), nulliparity (AOR: 1.30, 95% CI:1.04 -1.62), husbands with >10 years of education (AOR: 1.40, 95% CI: 1.09 - 1.81), being in the highest wealth quintile (AOR: 1.49, 95% CI: 1.18 - 1.89). For facility delivery, women's age, parity, women's education, husband's education, and wealth index were found to be determinants. None of the available socio-demographic factors were associated with four timely antenatal care visits.<br>Conclusions: The study observed socio-demographic inequalities associated with increased utilization of timely first antenatal care visit and facility delivery. The majority of women did not utilize one type of health facility for their antenatal care visits and delivery. Maternal health care programs should prioritize women who have husbands with low education, are of low socioeconomic status, young age, and multiparity to increase the health service utilization and promote good health.
**Order of Authors:**	Jesmin Pervin
	Mahima Venkateswaran
	U Tin Nu
	Monjur Rahman
	Brian F. O'Donnell
	Ingrid K. Friberg
	Anisur Rahman
	J. Frederik Frøen
**Additional Information:**	
**Question**	**Response**

Powered by Editorial Manager® and ProduXion Manager® from Aries Systems Corporation

**Financial Disclosure**<br><br>Enter a financial disclosure statement that describes the sources of funding for the work included in this submission. Review the submission guidelines for detailed requirements. View published research articles from *PLOS ONE* for specific examples.<br><br>This statement is required for submission and **will appear in the published article** if the submission is accepted. Please make sure it is accurate.<br><br>**Unfunded studies**<br>Enter: *The author(s) received no specific funding for this work.*<br><br>**Funded studies**<br>Enter a statement with the following details:<br>• Initials of the authors who received each award<br>• Grant numbers awarded to each author<br>• The full name of each funder<br>• URL of each funder website<br>• Did the sponsors or funders play any role in the study design, data collection and analysis, decision to publish, or preparation of the manuscript?<br>• **NO** - Include this sentence at the end of your statement: *The funders had no role in study design, data collection and analysis, decision to publish, or preparation of the manuscript.*<br>• **YES** - Specify the role(s) played.<br><br>* typeset	This research is part of the eRegistries Bangladesh project funded by the Norwegian Research Council (grant agreement number 248073/H10; title: Strengthening the extension of Reproductive, Maternal, Newborn, and Child Health services in Bangladesh with an electronic health registry: A cluster randomized controlled trial), and the Centre for Intervention Science in Maternal and Child Health (CISMAC), Center for International Health, University of Bergen (project number: 223269). icddr,b is also grateful to the Governments of Bangladesh, Canada, Sweden and the UK for providing core/unrestricted support. The funders had no role in the design of the study and collection, analysis, and interpretation of data.
**Competing Interests**<br><br>Use the instructions below to enter a competing interest statement for this submission. On behalf of all authors, disclose any competing interests that could be perceived to bias this work—acknowledging all financial support and any other relevant financial or non-financial competing interests.<br><br>This statement is required for submission	No authors have competing interests.

Powered by Editorial Manager® and ProduXion Manager® from Aries Systems Corporation

and **will appear in the published article** if the submission is accepted. Please make sure it is accurate and that any funding sources listed in your Funding Information later in the submission form are also declared in your Financial Disclosure statement.<br><br>View published research articles from *PLOS ONE* for specific examples.<br><br>**NO authors have competing interests**<br><br>Enter: *The authors have declared that no competing interests exist.*<br><br>**Authors with competing interests**<br><br>Enter competing interest details beginning with this statement:<br><br>*I have read the journal's policy and the authors of this manuscript have the following competing interests: [insert competing interests here]*<br><br>* typeset	
**Ethics Statement**<br><br>Enter an ethics statement for this submission. This statement is required if the study involved:<br><br>• Human participants<br>• Human specimens or tissue<br>• Vertebrate animals or cephalopods<br>• Vertebrate embryos or tissues<br>• Field research<br><br>Write "N/A" if the submission does not require an ethics statement.<br><br>General guidance is provided below. Consult the submission guidelines for detailed instructions. **Make sure that all information entered here is included in the Methods section of the manuscript.**	The study was approved by the Research and Ethical Review Committees of the International Centre for Diarrhoeal Disease Research, Bangladesh, and the Regional Ethical Committee in Norway, Southeast region. All participants received an explanation of the purpose of the study and gave written informed consent for participation in the study.

**Format for specific study types**<br><br>**Human Subject Research (involving human participants and/or tissue)**<br>• Give the name of the institutional review board or ethics committee that approved the study<br>• Include the approval number and/or a statement indicating approval of this research<br>• Indicate the form of consent obtained (written/oral) or the reason that consent was not obtained (e.g. the data were analyzed anonymously)<br><br>**Animal Research (involving vertebrate animals, embryos or tissues)**<br>• Provide the name of the Institutional Animal Care and Use Committee (IACUC) or other relevant ethics board that reviewed the study protocol, and indicate whether they approved this research or granted a formal waiver of ethical approval<br>• Include an approval number if one was obtained<br>• If the study involved *non-human primates*, add *additional details* about animal welfare and steps taken to ameliorate suffering<br>• If anesthesia, euthanasia, or any kind of animal sacrifice is part of the study, include briefly which substances and/or methods were applied<br><br>**Field Research**<br><br>Include the following details if this study involves the collection of plant, animal, or other materials from a natural setting:<br>• Field permit number<br>• Name of the institution or relevant body that granted permission	
**Data Availability**<br><br>Authors are required to make all data underlying the findings described fully available, without restriction, and from the time of publication. PLOS allows rare exceptions to address legal and ethical concerns. See the PLOS Data Policy and FAQ for detailed information.	Yes - all data are fully available without restriction

Powered by Editorial Manager® and ProduXion Manager® from Aries Systems Corporation

A Data Availability Statement describing where the data can be found is required at submission. Your answers to this question constitute the Data Availability Statement and **will be published in the article**, if accepted.<br><br>**Important:** Stating 'data available on request from the author' is not sufficient. If your data are only available upon request, select 'No' for the first question and explain your exceptional situation in the text box.<br><br>Do the authors confirm that all data underlying the findings described in their manuscript are fully available without restriction?	
**Describe where the data may be found in full sentences. If you are copying our sample text, replace any instances of XXX with the appropriate details.**<br><br>• If the data are **held or will be held in a public repository**, include URLs, accession numbers or DOIs. If this information will only be available after acceptance, indicate this by ticking the box below. For example: *All XXX files are available from the XXX database (accession number(s) XXX, XXX.).*<br>• If the data are all contained **within the manuscript and/or Supporting Information files**, enter the following: *All relevant data are within the manuscript and its Supporting Information files.*<br>• If neither of these applies but you are able to provide **details of access elsewhere**, with or without limitations, please do so. For example:<br><br>*Data cannot be shared publicly because of [XXX]. Data are available from the XXX Institutional Data Access / Ethics Committee (contact via XXX) for researchers who meet the criteria for access to confidential data.*<br><br>*The data underlying the results presented in the study are available from (include the name of the third party*	All relevant data are available within the manuscript and its supporting Information file.

and contact information or URL).<br>• This text is appropriate if the data are owned by a third party and authors do not have permission to share the data.<br><br>* typeset	
Additional data availability information:	

Powered by Editorial Manager® and ProduXion Manager® from Aries Systems Corporation

Manuscript Click here to access/download;Manuscript;Manuscript.doc

Determinants of utilization of antenatal and delivery care at the community level in rural Bangladesh

Jesmin Pervin [1,2], Mahima Venkateswaran [2,3], U Tin Nu[1], Monjur Rahman[1], Brian F. O'Donnell[3], Ingrid K. Friberg [3,4], Anisur Rahman [1], J. Frederik Frøen [2,3]

[1] International Centre for Diarrhoeal Disease Research, Bangladesh

[2] University of Bergen, Bergen, Norway

[3] Norwegian Institute of Public Health, Oslo, Norway

[4] Tacoma-Pierce County Health Department, Tacoma, WA, USA

*Correspondence to Dr. Jesmin Pervin; International Centre for Diarrhoeal Disease Research, Bangladesh; 68 Shaheed Tajuddin Ahmed Sarani; Mohakhali; Dhaka 1212; Bangladesh; Phone: 880-1713257397; E-mail: jpervin@icddrb.org

Abstract

Background: Timely utilization of antenatal care and delivery services supports the health of mothers and babies. This study aims to assess the utilization, timeliness of, and socio-demographic determinants of antenatal and delivery care services in two sub-districts in Bangladesh.

Methods: This cross-sectional study used data collected through a structured questionnaire in the eRegMat cluster-randomized controlled trial, which enrolled pregnant women between October 2018-June 2020. Bivariate and multivariate logistic regression were employed. To determine the associations of socio-demographic variables with timely first ANC, four timely ANC visits, and facility delivery, binary logistic regression was executed.

Results: Data were available on 3293 pregnant women. Attendance at a timely first antenatal care visit was 59%. Uptake of four timely antenatal care visits was 4.2%. About three-fourths of the women delivered at a health facility. Women from all socioeconomic groups gradually shifted to utilizing private hospitals from the public health facilities, as the pregnancy advanced. Timely first antenatal care visit was associated with: women over 30 years of age (AOR: 1.52, 95% CI: 1.05 - 2.20), nulliparity (AOR: 1.30, 95% CI:1.04 -1.62), husbands with >10 years of education (AOR: 1.40, 95% CI: 1.09 - 1.81), being in the highest wealth quintile (AOR: 1.49, 95% CI: 1.18 - 1.89). For facility delivery, women's age, parity, women's education, husband's education, and wealth index were found to be determinants. None of the available socio-demographic factors were associated with four timely antenatal care visits.

Conclusions: The study observed socio-demographic inequalities associated with increased utilization of timely first antenatal care visit and facility delivery. The majority of women did not utilize one type of health facility for their antenatal care visits and delivery. Maternal health care programs should prioritize women who have husbands with low education, are of low socioeconomic status, young age, and multiparity to increase the health service utilization and promote good health.

Trial registration: ISRCTN69491836. Registered on December 06, 2018. Retrospectively registered.

Keywords: Antenatal Care, Facility Delivery, Timely first ANC, Timely four ANC visits, Cross-sectional study

Introduction

Timely utilization of antenatal care (ANC) and delivery services is important to support maternal health and allow for the best possible start to babies' lives. Most pregnancy complications leading to severe morbidity or mortality can be prevented or treated if women use healthcare services provided by skilled providers with the necessary resources according to recommended clinical guidelines [1]. Utilization of a health facility during pregnancy allows for recognition of warning signs of complications, referral mothers to emergency care, and management of severe complications during pregnancy and childbirth. Despite progress in maternal health service utilization in some low- and middle-income countries, further increase in utilization is needed to impact maternal and neonatal health [2].

Bangladesh has decreased maternal and neonatal mortality remarkably from 1990 to 2018. Maternal mortality reduced from 570 to 196 per 100,000 live births during that period [3]. For the same period, neonatal mortality reduced from 59 to 30 per 1000 live births [4, 5]. However, the levels of maternal and neonatal mortality have remained stagnant since 2015, the end of Millennium Development Goal (MDG) era. Sustainable Development Goal 3 emphasizes the end of all preventable deaths and sets the target to reduce the maternal mortality ratio to less than 70 per 100,000 live births and neonatal mortality to at least as low as 12 per 1,000 live births by 2030 [6]. To achieve these targets, better health care utilization in both pregnancy and delivery are essential.

ANC utilization is a crucial step for timely identification, prevention, and management of factors influencing pregnancy outcomes [7-9]. While significant progress has been shown worldwide in the use of health facilities during childbirth (77%) [10], only about 64% of women receive four or more ANC visits [11, 12]. Late initiation of ANC is one reason for underutilization of care, subsequently leading to increased risk of adverse pregnancy outcomes [9, 11]. ~~Increased utilization of timely ANC services facilitates women and their family's introduction to the formal health system, promoting early risk identification and improving pregnancy outcomes.~~

In Bangladesh, the ~~utilization~~ of health facilities for ANC and childbirth has been steadily increasing over the years. About 47% of women receive four or more ANC visits. However, only 29% of women receive a first ANC visit before 16 weeks and 17% of pregnant women still do not receive any ANC. About half of all deliveries occur in health facilities, most of them privately-owned [5]. The use of public and non-governmental health facilities for childbirth has increased but to a lesser extent. There is considerable inequalities in the utilization of maternal health services in the different sub-districts of Bangladesh [13]. A recent cross-sectional study conducted in a sub-district of the Noakhali district found lower utilization of ANC services (34.6%) and facility delivery (5.3%) compared to reported national statistics [13].

Studies in Bangladesh have reported many factors that are associated with the use of facilities for ANC, such as age, religion, parity, having a living child, educational attainment of women, place of residence, household wealth status, decision-making power, complications during the current pregnancy or a previous pregnancy, husband's education, and access to mass media [14, 15]. Similar factors have been identified as determinants of institutional delivery [13, 16, 17].

The Government of Bangladesh recommends four focused ANC visits for all low-risk pregnancies based on 2002 WHO recommendations: first ANC visit (within 16+^{6} weeks of gestation), second ANC visit (24+^{0} to 28+^{6} weeks of gestation), third ANC visit (at 32+^{6} weeks of gestation) and fourth ANC visit (at 36+^{6}

weeks of gestation) [12]. Though utilization of care is increasing, few studies exist on either the utilization or determinants of timely ANC. Also, ~~information on the use of different types of health facilities for ANC and delivery care services at the community-level is unavailable. Information on the~~ utilization patterns of public, private, and non-government organization (NGO) health facilities, based on clients' socio-economic and demographic differences, is essential to effectively improve women's care-seeking during pregnancy and childbirth. The present study aims to assess the utilization, timeliness of, and socio-demographic determinants of antenatal and childbirth care services in two sub-districts in Bangladesh.

Methods

Study design

This cross-sectional study used data collected as part of a cluster-randomized controlled trial, eRegMat, conducted in two sub-districts, Matlab South and Matlab Nor[illegible]nder Chandpur district of Bangladesh (trial registration: ISRCTN69491836). Women with pregnancies identified and registered from October 2018 to June 2020 were enrolled in the eRegMat trial.

Study setting

The estimated population of Matlab South and Matlab North sub-districts are approximately 200,000 and 300,000, respectively [18]. Matlab South is divided into eight administrative blocks known as unions, while Matlab North has 14 unions. The Ministry of Health and Family Welfare (MOHFW) provides maternal and child health services through two divisions: the Directorate General of Family Planning (DGFP) and the Directorate General of Health Services (DGHS). Under the DGFP, each Union Health & Family Welfare Centre (UH&FWC) serves a population of 25,000. Family welfare visitors (FWV)

work at the UH&FWC. Family welfare assistants (FWA) provide community outreach services and are the first contacts of the population at the household level. Under the DGHS, one to four community clinics (CC) provide services in each union, serving about 6000 people each. Community health care providers (CHCP) serve at community clinics (CC), and health assistants (HA) provide care at the household level. Matlab Health and Research Centre, run by the International Centre for Diarrhoeal Disease Research, Bangladesh (icddr,b), and a few non-government organizations (NGOs) also provide maternal and child health care.

Since 2018, a digital maternal and child health registry (eRegistry) has been implemented in two sub-districts for use by both CHCP and FWV in health facilities and by HA and FWA for community-level services. The eRegistry is designed so both facility-based and community-based health workers can access an individual's client record and input clinical data. All pregnancies are supposed to be registered in the eRegistry so as to create comprehensive client records. In total, 72 health facilities were included in the eRegistry roll-out, and a cluster-randomized controlled trial (eRegMat) was embedded in the implementation. In health facilities assigned to the intervention group (n=30), three digital health interventions were implemented in addition to the digital longitudinal tracking – clinical decision support, feedback dashboards for health workers, and targeted client communication via SMS to pregnant women. The control group (n=29) facilities used an eRegistry without additional digital health interventions. Pregnancy registrations in a randomized health facility were automatically allocated to their respective intervention or control group, while community registrations received a trial allocation based on the woman's choice of health facility for ANC. Health facilities that were not included in the trial received the eRegistry without digital health interventions to maintain continuity of data and care in the health system and were classified as non-randomized (n=13).

For this analysis, we included data on all women in the control and non-randomized groups of the eRegMat trial, as well as women registered in the eRegistry by community-level health workers without trial allocation. Women randomized to the intervention group were excluded in this analysis.

Data availability

Data were collected from women within eight to fourteen days after childbirth. For a few cases, the data collection period was extended up to nine months, either because women were not available within seven to fourteen days after childbirth or due to the COVID-19 pandemic lockdown measures. Written consent was obtained for the postpartum survey during pregnancy registration in the eRegistry. A structured questionnaire was prepared for the survey; the questionnaire was pretested before data collection began. Data were collected on utilization of ANC and delivery services, birth outcomes, and respondents' socio-economic characteristics. Twenty female data collectors with experience collecting data in household surveys from the same community were recruited and trained for data collection. Two data collectors were appointed to call pregnant women every other week after 28 weeks of gestation and through 35 weeks of gestation and then once a week until their delivery. A monitoring dashboard was developed to identify pregnant women for phone calls based on the gestational age in the eRegistry and collect their pregnancy outcome information. From the monitoring dashboard, one field research assistant produced lists of enrolled women who had a pregnancy outcome daily and distributed those lists to the data collectors. Data collectors then visited the women to conduct the interview after childbirth. After data collection, the survey questionnaires were checked for completeness and discrepancies by the data collectors' supervisors. Data were entered into a web-based electronic form by assigned data entry staff.

Outcome variables

The outcome variables and definitions were as follows: 1) timely first ANC visit: a visit within $17+^{6}$ weeks of gestation; 2) four timely ANC visits: ANC visits at or before $17+^{6}$ weeks, $24+^{0}$ to $28+^{6}$ weeks, $31+^{0}$ to $33+^{6}$ weeks and $35+^{0}$ to $37+^{6}$ weeks of gestation according to the national ANC schedule [19]; and 3) facility delivery: a delivery in any health facility, including public, private, and those run by icddr,b and NGOs. To allow for maternal opportunity to attend ANC, we expanded the specific weeks by an additional one-week range, except when the guideline already recommended a range of weeks (for example, $24+^{0}$ to $28+^{6}$ weeks), where we kept the original range.

We also analyzed the associations between sociodemographic determinants and Skilled Birth Attendant (SBA) at delivery. SBA at delivery were considered if delivery was conducted by qualified doctor, nurse, midwife, paramedic, family welfare visitor (FWV) or community skilled birth attendant (CSBA) as described in the Bangladesh Health and Demographic Survey [5].

Explanatory Variables

The predictor variables considered in the analysis were age of women, parity, women's education, husband's education, and household wealth index. Parity was defined as the number of times that a woman has given birth to a baby with a gestational age of 28 weeks or more, regardless of whether it was a live birth or stillbirth. Educational status was assessed as the number of completed years of schooling. Household wealth index was calculated by generating scores through principal-components analysis based on household assets of ownership of a number of consumer items (freezer, television, etc.), household livestock, dwelling characteristics (wall and roof material), type of drinking water, toilet facilities, type of fuel mainly used for cooking, and source of electricity. These scores were then indexed into quintiles, where one represented the lowest and five the highest. The last menstrual period date

was determined by recall during the interview at the household visit, for consistency. Gestational age at each ANC visit was measured by subtracting the LMP date from the ANC visit date and expressed in weeks and days.

Sample size

The total available sample size was 3293 pregnancies. Power calculations were made on the study outcomes. According to the Bangladesh health and demographic survey report and a recent study conducted in Bangladesh we could expect 37% to attend a first timely ANC, 1% to attend four timely ANC visits and 50% of women to have a facility delivery during the study period [5, 20]. To estimate whether our available sample size was sufficient for the detection of prevalence we calculated the power to estimate bounds on the most conservative estimate of 50% of the facility delivery with 4% error probabilities, and an α of .05. The power was estimated at 93%, well above the levels needed, thus our proposed sample size was sufficient to measure the prevalence of timely first ANC, four timely ANC and facility delivery and their association with sociodemographic determinants. We used Stata version 16 for the power calculations.

Data analysis

We used descriptive statistics to present women's socio-demographic characteristics and the utilization of antenatal and delivery care using percentage distribution, mean, and median. Bivariate analysis was employed to determine the associations between each of the dependent variables and the selected explanatory variables. The level of association was tested by Pearson's chi-square (χ2) tests and statistical significance was considered if the p-value was found to be <0.05. Maternal age was categorized into <20, 20 – 24, 25 – 34, ≥35 years, parity into 0, 1, ≥2, and education into 0– 5, 6 – 10, > 10 years of schooling Quintiles of asset scores were used to categorize socio-economic status.

A first ANC visit was considered to be timely if care was received within week 17+^{6} of gestational age. All other first ANC visits were considered not timely. Similarly, the other three routine ANC visits were defined timely if they occurred between week 24+^{0} to 28 +^{6}, week 31+^{0} to 33+^{6}, and week 35+^{0}to 37+^{6}. Women were considered to have completed four timely ANC visits if one timely ANC visit was received within each of the four recommended periods of time; all other ANC visits were considered not timely. To determine the associations of socio-demographic variables with timely first ANC, four timely ANC visits, and facility delivery, binary logistic regression was executed. The results of the binary logistic regression analyses were presented by odds ratios (OR) with 95% confidence intervals (CI). All the socio-demographic variables related to the outcomes of interest were included in the multivariate model to adjust for potential confoun[illegible]g. All statistical analyses were done in Stata version 16 [21].

Results

Socio demographic and economics characteristics

We included a total of 3293 women in the analysis. Of all the women, 84% were interviewed within 8-14 days of childbirth. The characteristics of the women are shown in Table 1. The participants' mean age was 24 years (SD ±4.5); about 18% of women were under the age of 20 years. The median parity was 2, and 40% of participants were nulliparous, while one-fourth of the participants had 2 or more children. The median number of years of school attendance for study participants and their husbands was 9 and 8 years, respectively.

ANC Utilization

Almost all participants (98%) received at least one ANC visit, while 91% of women received two visits, 74% received three visits and half of the participants (52%) received four or more ANC visits. The mean

gestational age (GA) at first, second, third and fourth ANC visit was 17.6 weeks (SD ±6.9), 24.3 weeks (SD ±6.8), 28.4 weeks (SD± 5.9) and 31.1 weeks (±4.9) respectively. More than half of the participants (59%) attended a timely first ANC visit and 62%, 42% and 31% of women received timely 2^{nd,} 3rd and 4th ANC visits, respectively. Overall, 94% received one ANC visit timely, 68% attended two ANC visits timely and 22% utilized three ANC visits timely. But only 4.2% of women attended all four ANC visits according to the recommended ANC schedule.

Among the women who attended at least one ANC visit (Fig 1), about 45% attended their first ANC visit in public health facilities, whereas 41% of women visited private facilities. On the other hand, 51% of women received their 4th ANC visit in private facilities, and only 32% in public facilities.

Fig 1. Place of ANC utilization among all women

For ANC visits after the first visit (Fig 2), the public health sector gradually lost more women compared to the private, and icddr,b and NGO health facilities. Of the women who visited a public health facility for their first ANC visit, 56%, 42%, and 26%, used public health facilities for their second, third and fourth ANC visits and 17% for childbirth. Of the women who visited a public health facility for their first ANC visit, 51% of them attended private health facilities for delivery.

Of the women (1445) who received their first ANC visit from public facilities, only 6.3% visited public health facilities for all four ANC visits. Among women (1315) who received their first ANC from private facilities, 5.2% received four ANC visits from private facilities. In the case of women (n=398) who received their first ANC visit from icddr,b and NGOs, 3.8% of women went to NGO facilities for all four ANC visits.

Fig 2. Patterns of health facility use for subsequent antenatal and delivery care based on the place of the first antenatal care visit.

Most women received their first ANC visit from doctors (Fig 3). Similar trends were seen for the type of healthcare provider for all four ANC visits (Fig 3). Doctors who provided first ANC were mostly (92%) from private facilities, while 89% of nurses and midwives were from NGOs and icddr,b.

Fig 3. ANC utilization by health care providers. CHW (Community Health Workers: FWA, HA, others; FWV (Family Welfare Visitor); CHCP (Community Health Care Provider).

Facility delivery

Among all respondents (n=3293), 74% of women delivered at a health facility. Of the women who gave birth at a facility, 75% delivered at private facilities, 19% at public health facilities, and 6% at icddr,b, and NGO-led facilities. More than half of the deliveries (54%) were conducted by doctors (Fig 4); 92% of the doctors were from private facilities. About 80% of women used skilled birth attendants (SBA) during childbirth. Of the women who delivered their baby by normal vaginal birth, 41% of them were conducted by a Traditional Birth Attendant (TBA). About half of the participants (51%) delivered their baby by caesarian section and 92% of the cesarean sections occurred in private facilities.

Fig 4. Type of provider performing delivery.

Socio-economic characteristics, and utilization of ANC and facility delivery

During the first ANC visit (Fig 5), 55% of women from the poorest group and 36% from the richest group utilized public facilities, whereas 32% of the poorest group and 49% of the richest group used private health facilities. In the case of icddr,b, and NGO-led facilities, utilization was 10.5% and 13% among the

poorest and richest groups, respectively. For the 4th ANC visit and delivery, private health facilities were preferentially utilized by all socio-economic groups (Figs 5 and 6).

Fig 5. Health facility utilization for timely ANC visits by wealth index.

Fig 6. Health facility utilization for facility delivery by wealth index.

Socio-economic determinants of timely first ANC visit, four timely ANC visits and facility delivery

Women's age, parity, husband's education, and socioeconomic status were associated with a timely first ANC visit (Table 1). Women more than 30 years of age were 1.5 times more likely to attend a timely first ANC visit compared to women less than 20 years of age. Nulliparous women were 1.3 times more likely to attend a timely first ANC visit compared to women with two or more births. Women whose husbands had completed more than ten years of education were 40% more likely to attend a timely first ANC visit than if their husbands had 0-5 years of schooling. Women with higher socioeconomic status were more likely to have a timely first ANC visit. We did not find any associations between the socio-demographic determinants used in our analysis and four timely ANC visits (Table 1).

Women's age, parity, women's education, husbands' education, and socio-economic status were associated with facility delivery (Table 1). Women 30 years or older were 1.6 times more likely to deliver in the health facility than women less than 20 years of age. Nulliparous women were 1.5 times more likely to use a health facility for childbirth compared to women with two or more births. Women who completed more than 10 years of school were 97% more likely to use the health facility for childbirth in comparison to women with less than 5 years of education. Women were 53% more likely to have a

facility delivery if their husbands completed more than 10 years of school than 0-5 years of education. Women belonging to the highest group of wealth index were 2.2 times more likely to deliver in the health facility. Results with unadjusted odds ratios are presented in a supplementary file (S1 Appendix). Similar associations were found for SBA as for facility delivery (data not shown, in S2 Appendix).

Table 1: Associations of timely first ANC visit, timely four ANC visit, and facility delivery with socio-demographic determinants

Characteristics (n=3293)	N (%)	Timely first ANC visit (n=3242)		Timely four ANC visits (n=3242)		Facility delivery (n=3293)	
		n (%)	Adjusted OR*(95% CI)	n (%)	Adjusted OR* (95% CI)	n (%)	Adjusted OR* (95% CI)
Age in years							
<20	581 (18)	339 (59)	1	20 (5.1)	1	437 (75)	1
20-30	2470 (75)	1433 (59)	1.16 (.93 - 1.45)	103 (6.0)	1.07 (.60 - 1.93)	1826 (74)	1.14 (.88 - 1.48)
>30	242 (7)	148 (63)	1.52 ([illegible] - 2.19)	13 (7.4)	1.21 (.52 - 2.86)	183 (76)	1.60 (1.05 - 2.43)
Parity							
0	1145 (35)	653 (58)	1.30 (1.04 - 1.62)	47 (6.0)	.68 (.40 - 1.16)	860 (75)	1.52 (1.18 - 1.95)
1	1306 (40)	804 (62)	1.06 (.88 - 1.29)	47 (5.0)	.79 (.50 - 1.25)	1014 (78)	1.38 (1.12- 1.71)
≥ 2	842 (26)	463 (57)	1	42 (7.5)	1	572 (68)	1
Years of education							
0-5	426 (13)	230 (56)	1	12 (4.2)	1	260 (61)	1
6-10	2285 (69)	1302(58)	.94 (.75 - 1.18)	98 (6.3)	1.57 (.83 - 2.97)	1693 (74)	1.38 (1.09 - 1.74)
>10	582 (18)	388 (67)	1.13 (.84 - 1.52)	26 (5.8)	1.46 (.66 - 3.21)	493 (85)	1.97 (1.40 - 2.77)
Husband education							
0-5	888 (27)	464 (54)	1	36 (6.3)	1	577 (65)	1
6-10	1879 (57)	1106 (60)	1.14 (.96 - 1.36)	69 (5.2)	.78 (.51 - 1.21)	1433 (76)	1.31 (1.09 - 1.58)
>10	526 (16)	350(67)	1.40 (1.09 - 1.81)	31 (7.7)	1.25 (.71 - 2.19)	436 (83)	1.53 (1.14 - 2.06)
Assets							
Lowest	671 (20)	336 (52)	1	18 (4.3)	1	422 (63)	1
Second	669 (20)	372 (57)	1.17 (.94 - 1.46)	25 (5.8)	1.40 (.75 - 2.60)	467 (70)	1.23 (.98 - 1.56)
Middle	746 (23)	463 (62)	1.45 (1.17 - 1.81)	35 (6.5)	1.71 (.95- 3.08)	585 (78)	1.84 (1.44 - 2.34)
Fourth	552 (17)	329 (60)	1.27 (1.00 - 1.62)	27 (6.8)	1.74 (.93 -3.26)	428 (78)	1.64 (1.26 - 2.14)
Highest	655 (20)	420 (65)	1.49 (1.18 - 1.89)	31 (6.3)	1.67 (.89 -3.11)	544 (83)	2.15 (1.63 - 2.82)

*Adjusted with age, parity, women's education, husband's education, and assets

Discussion

The study found six in ten women attended a timely first antenatal care visit, but that utilization of four timely ANC visits was very low. Seven in ten women delivered at a health facility. Our study also found that women gradually moved to private hospitals from earlier use of public health facilities, as the pregnancies advanced. The study identified socioeconomic inequities in public versus private health facilities for utilization of ANC and childbirth care. We identified age, parity, wealth index, and education of women's husband as determinants of timely first ANC visit and facility delivery.

Utilization of private hospitals for maternal healthcare services is increasing all over the Bangladesh. The unavailability of essential maternal healthcare supplies, services, care providers and the interest of dual practice doctors in the public sector might encourage women to resort to more expensive private sector healthcare [22]. Similar trends were observed in other studies from low- and- middle income countries [23, 24]. Women's negative perceptions and experiences of public health care could also drive them to private health facilities and away from public health facilities [23, 25]. Women and family members' preference to receive care from doctors, especially during the last trimester of pregnancy and for delivery, might drive care-seeking toward private health facilities. Our study c~~onfirmed~~ that, of the women receiving care from doctors, the majority were from private hospitals. ~~The Bangladesh Demographic and Health Survey (DHS) also reported that a majority of women who used a health facility for childbirth delivered at a private clinic~~ [5]. A study conducted in Bangladesh reported increased utilization of private health facilities compared to public health facilities for maternal health care in general [25, 26]. Basic and comprehensive packages of emergency obstetric care with skilled providers, ~~especially doctors~~, may need to routinely be available throughout the public sector to increase retention of women who start their ANC in hopes that they remain there for delivery care.

The coverage of a timely first ANC visit in our study is higher than reported in a study conducted in three northern districts of Bangladesh, where 32% of women received a timely first ANC visit from a skilled provider [20]. Our study also shows somewhat higher coverage of a timely first ANC visit compared to the national coverage reported in the most recent DHS (37%) [5]. Another study conducted in Bangladesh measuring initiation of ANC, as a first visit within 12 weeks of gestation, also found low coverage [27]. Implementation of the Maternal Healthcare Voucher program (DSF) in this area by the Ministry of Health and Family Welfare in Bangladesh since 2010, might explain the higher coverage in our study compared to national reports. Several studies have reported low levels of early initiation of ANC in Asia and Africa [28-31], while others have reported higher coverage of an ANC visit within 16 weeks [32-34], suggesting wide within- and between-country variations.

Although half of women received four or more ANC visits, our study found low coverage of four timely ANC visits illustrating that women might attend adequate numbers of visits, but they lacked actionable information on the nationally recommended ANC visits schedule. The coverage of four timely ANC visits was found to be low despite the higher utilization of the first timely ANC. A possible explanation could be women visited the public health facility for their first ANC as the health facility was very close to their house. They were not oriented then about the availability of services. By the time of the fourth visit, they were often trying to get more essential services such as ultrasonogram which is available in the private facilities. Other studies conducted in northern Bangladesh and Ethiopia found low coverage of four timely ANC visits despite high uptake of the first ANC timely [20, 35]. In Ethiopia, though their first ANC coverage is less than ours, their timely four ANC utilization was notably greater than found in this study. This illustrates that the drivers of timely first ANC and timely four ANC visits may differ within countries and may differ between countries as well. More information is needed to understand these differences. We need to assess the context-specific mechanisms to increase the four timely ANC

utilization. Use reminders by phone call or text message could be employed to increase the timeliness of ANC attendance[36-38].

Our results provide additional supportive evidence that institutional delivery (74%) is still increasing, including in this particular area. A survey conducted in 2015 in our study area reported a coverage of 49% [39]. The higher coverage in our results may be attributed to the implementation of several interventions to increase facility delivery in the study area, including strengthened health education activities by health workers [40]. Demand side financing for ultra-poor women is another such intervention [41, 42]. Other reasons might include increased availability of and access to health facilities as well as overall improvements in the population's economic status [43, 44].

Women's husbands' education appears to play a critical role in initiating early ANC and facility delivery. In Bangladesh, men often have the privilege to make decisions for their wives, which may explain the association between husbands' education and maternity service utilization [16, 17, 31, 45]. Our results also show that more educated women were more likely to have an institutional delivery. This may be attributed to the fact that women with higher education have a better understanding of seeking care, more awareness of the value of health care utilization, and making decisions with confidence ~~[16, 28, 30, 34, 46-49]~~.

This study also found that women's economic status was a crucial predictor of compliance with the nationally recommended first ANC contact and facility delivery, with the richest women more likely to seek early health care. Our results are similar to previous studies that found a positive association between the economic status of women and early initiation of ANC and facility delivery, probably due to ease of access to health care [44, 48, 50]. This study also identified disparities in the use of public and private health facilities for ANC and childbirth care services in Bangladesh's rural context. Poor women used public health facilities more, while rich women were more likely to use private facilities. Designing

interventions based on inequities in maternal health service utilization is important to increase access to those actually need support.

This study has several strengths. Our study is a large, cross-sectional population-based study. Women were interviewed within a short period after birth potentially minimizing the magnitude of the recall bias. Most studies of maternal healthcare utilization and coverage have used survey data, where women with a live birth in the two-three years before the survey were interviewed, which might introduce recall bias. We used a standardized questionnaire to ensure comparable responses from the participants. We used an asset-based index, which is a good proxy for measuring household wealth status. One of the limitations of the study lies in the accurate assessment of gestational age at the ANC visit. Women's recall of the dates of ANC visits could be incomplete or faulty when such data are collected retrospectively through household surveys [51]. This was available for a subset of women through the eRegistry, but we chose to use the data from recall for consistency. Unmeasured predisposing, enabling, and need-based factors not included in our analysis might affect coverage of timely first ANC, timely four ANC visits, and facility delivery. Coverage of four timely visits was low, which precluded meaningful analysis of associations.

The study was conducted to assess in detail patterns of health facility utilization in Matlab. Our evidence suggested that women might shift frequently between public and private health system, possibly to get better quality of care. Continuity of care helps to improve the quality of care by establishing good relationships and trust between the provider and women. [52, 53]. A comprehensive description of women’s’ transition between facilities and types of facilities is critical background to understanding women’s decision-making processes and reasoning and is necessary to design appropriate health care interventions to improve care seeking quantity, continuity and timeliness. Further exploration is required to explain their choices and to attempt to encourage continuity of information during

pregnancy and delivery. Remaining within a single health system is likely to improve the probability of increased timeliness of ANC utilization.

Maternal health care programs should prioritize women with less educated husbands, low socio-economic condition, young age, and multiparity to increase the timely health service utilization and promote good health. Further exploration is required to understand the drivers and needs of these women to increase timely health care utilization. Innovative interventions appropriate for the local settings to address these gaps and increase timely maternal service utilization are crucial.

Abbreviations

ANC: Antenatal care; **AOR:** Adjusted Odds Ratio; **eRegMat**: electronic registry Matlab; **DHIS2:** District Health Information System 2; **CC**: Community Clinic; **UH&FWC:** Union Health and Family Welfare Centres; **MOHFW:** Ministry of Health and Family Welfare; **DGFP**: Directorate General of Family Planning; **DGHS**: Directorate General of Health Services; **FWV:** Family Welfare Visitor; **FWA:** Family Welfare Assistant; **CHCP:** Community Health Care Provider; **HA:** Health Assistant; **NGO:** Non-government organization; **icddr,b :** International Centre for Diarrheal Disease, Bangladesh

Declarations

Ethics approval and consent to participate

The study was approved by the Research and Ethical Review Committees of the International Centre for Diarrhoeal Disease Research, Bangladesh, and the Regional Ethical Committee in Norway, Southeast region. All participants received an explanation of the purpose of the study and gave written informed consent for participation in the study.

Consent for publication

Not applicable.

Availability of data and materials

All relevant data are available within the manuscript and its supporting Information file.

Funding

This research is part of the eRegistries Bangladesh project funded by the Norwegian Research Council (grant agreement number 248073/H10; title: Strengthening the extension of Reproductive, Maternal, Newborn, and Child Health services in Bangladesh with an electronic health registry: A cluster randomized controlled trial), and the Centre for Intervention Science in Maternal and Child Health (CISMAC), Center for International Health, University of Bergen (project number: 223269). icddr,b is also grateful to the Governments of Bangladesh, Canada, Sweden and the UK for providing core/unrestricted support. The funders had no role in the design of the study and collection, analysis, and interpretation of data.

Authors' contributions

JFF, AR, IKF, JP, and MV contributed to the study concept and design. AR, IKF and JP supervised the implementation of the study. JP, UTN, and MR were involved in data collection and JP, MV, UTN, MR, BO, IKF, AR, and JFF contributed to data analyses. JP drafted the initial draft of the manuscript. JP, MV, UTN, MR, BO, IKF, AR, and JFF, reviewed and approved the final version of the manuscript.

Competing interests

The authors declare that they have no competing interests.

Acknowledgements

We would like to forward our gratitude to all the respondents who participated in this study.

References

1. Singh R, Neogi SB, Hazra A, Irani L, Ruducha J, Ahmad D, et al. Utilization of maternal health services and its determinants: a cross-sectional study among women in rural Uttar Pradesh, India. J Health Popul Nutr. 2019;38(1):13.
2. Geller SE, Koch AR, Garland CE, MacDonald EJ, Storey F, Lawton B. A global view of severe maternal morbidity: moving beyond maternal mortality. Reproductive Health. 2018;15(1):98.
3. NIPORT. Bangladesh Maternal Mortality and Health Care Survey 2016: Preliminary Report. Dhaka, Bangladesh; 2017
4. National Institute of Population Research and Training (NIPORT) MaAM, and ORC Macro (ORCM). Bangladesh Demographic and Health Survey 1999-2000. 2001.
5. NIPORT. Bangladesh Demographic and Health Survey 2017-18: Key Indicators. Dhaka, Bangladesh, and Rockville: Maryland, USA: NIPORT, and ICF; 2019.
6. WHO. Health in 2015: from MDGs, Millennium Development Goals to SDGs, Sustainable Development Goals. 2015.
7. Pervin J, Moran A, Rahman M, Razzaque A, Sibley L, Streatfield PK, et al. Association of antenatal care with facility delivery and perinatal survival - a population-based study in Bangladesh. BMC Pregnancy Childbirth. 2012;12:111.
8. Pervin J, Rahman SM, Rahman M, Aktar S, Rahman A. Association between antenatal care visit and preterm birth: a cohort study in rural Bangladesh. BMJ Open. 2020;10(7):e036699.
9. WHO. WHO recommendations on antenatal care for a positive pregnancy experience. 2016.
10. UNICEF. Delivery care 2020 [Available from: https://data.unicef.org/topic/maternal-health/delivery-care/.
11. UNICEF. Antenatal Care 2019 [Available from: https://data.unicef.org/topic/maternal-health/antenatal-care/#:~:text=Current%20status%20%2B%20progress,-There%20are%20wide&text=Currently%20available%20data%20indicate%20that,at%20least%20four%20antenatal%20visits.
12. WHO. WHO antenatal care randomized trial : manual for the implementation of the new model. 2002.
13. Kabir MR, Ghosh S, Al Mamun MA, Islam H, Binta A Ghani R. Factors associated with antenatal and health facility delivery care in selected areas of Subornochor upazila, Noakhali, Bangladesh. Clinical Epidemiology and Global Health. 2020;8(3):983-8.

14. Chanda SK, Ahammed B, Howlader MH, Ashikuzzaman M, Shovo T-EA, Hossain MT. Factors associating different antenatal care contacts of women: A cross-sectional analysis of Bangladesh demographic and health survey 2014 data. PLOS ONE. 2020;15(4):e0232257.
15. Rahman A, Nisha MK, Begum T, Ahmed S, Alam N, Anwar I. Trends, determinants and inequities of 4(+) ANC utilisation in Bangladesh. J Health Popul Nutr. 2017;36(1):2-.
16. Kamal SM, Hassan CH, Alam GM. Determinants of institutional delivery among women in Bangladesh. Asia Pac J Public Health. 2015;27(2):Np1372-88.
17. Yaya S, Bishwajit G, Ekholuenetale M. Factors associated with the utilization of institutional delivery services in Bangladesh. PLOS ONE. 2017;12(2):e0171573.
18. BBS. Population and housing census-2011,National Volume-2: Union statistics. 2014.
19. (MOHFW) MoHaFW. Union Healh and Family Welfare Centre Guideline. 2014.
20. Sarker BK, Rahman M, Rahman T, Rahman T, Khalil JJ, Hasan M, et al. Status of the WHO recommended timing and frequency of antenatal care visits in Northern Bangladesh. PLOS ONE. 2020;15(11):e0241185.
21. StataCorp. Stata 16 Base Reference Manual. College Station, TX. Stata Press. 2019.
22. Roy A, Shengelia L. An Analysis on Maternal Healthcare Situation in Bangladesh: A Review. Diversity & Equality in Health and Care. 2016;13.
23. Rahman R. The State, the Private Health Care Sector and Regulation in Bangladesh. Asia Pacific Journal of Public Administration. 2014;29:191-206.
24. Prasad S. Preference of hospital usage in India. Annals of Tropical Medicine and Public Health. 2013;6(4):472-8.
25. Andaleeb S. Public and private hospitals in Bangladesh: Service quality and predictors of hospital choice. Health policy and planning. 2000;15:95-102.
26. Anwar I, Nababan HY, Mostari S, Rahman A, Khan JAM. Trends and Inequities in Use of Maternal Health Care Services in Bangladesh, 1991-2011. PLOS ONE. 2015;10(3):e0120309.
27. Siddique MA, Perkins J, Mazumder T, Haider MR, Banik G, Tahsina T, et al. Antenatal care in rural Bangladesh: Gaps in adequate coverage and content. PLoS ONE. 2018;13:e0205149.
28. Agha S, Tappis H. The timing of antenatal care initiation and the content of care in Sindh, Pakistan. BMC Pregnancy and Childbirth. 2016;16(1):190.
29. Baral R, Fleming J, Khan S, Higgins D, Hendrix N, Pecenka C. Inferring antenatal care visit timing in low- and middle-income countries: Methods to inform potential maternal vaccine coverage. PLOS ONE. 2020;15(8):e0237718.
30. Jiwani SS, Amouzou-Aguirre A, Carvajal L, Chou D, Keita Y, Moran AC, et al. Timing and number of antenatal care contacts in low and middle-income countries: Analysis in the Countdown to 2030 priority countries. J Glob Health. 2020;10(1):010502.
31. Tufa G, Gayesa R, Seyoum D. Factors Associated with Timely Antenatal Care Booking Among Pregnant Women in Remote Area of Bule Hora District, Southern Ethiopia. International Journal of Women's Health. 2020;Volume 12:657-66.
32. Abebo T, Anshebo A. Assessment of Timing of First Antenatal Care Booking and Associated Factors among Pregnant Women who attend Antenatal Care at Health Facilities in Dilla town, Gedeo Zone, Southern Nations, Nationalities, and Peoples Region, Ethiopia, 2014. Journal of Pregnancy and Child Health. 2016;3:258.
33. Gudayu TW. Proportion and Factors Associated with late Antenatal Care Booking among Pregnant Mothers in Gondar Town, North West Ethiopia. Afr J Reprod Health. 2015;19(2):94-100.
34. Paudel YR, Jha T, Mehata S. Timing of First Antenatal Care (ANC) and Inequalities in Early Initiation of ANC in Nepal. Frontiers in Public Health. 2017;5:242.

35. Ftwi M, Gebretsadik GG-e, Berhe H, Haftu M, Gebremariam G, Tesfau YB. Coverage of completion of four ANC visits based on recommended time schedule in Northern Ethiopia: A community-based cross-sectional study design. PLOS ONE. 2020;15(8):e0236965.
36. Feroz AS, Perveen S, Aftab W. Role of mHealth applications for improving antenatal and postnatal care in low and middle income countries: A systematic review. BMC Health Services Research. 2017;17.
37. Labrique AB, Wadhwani C, Williams KA, Lamptey P, Hesp C, Luk R, et al. Best practices in scaling digital health in low and middle income countries. Globalization and Health. 2018;14(1):103.
38. Lund S, Nielsen BB, Hemed M, Boas IM, Said A, Said K, et al. Mobile phones improve antenatal care attendance in Zanzibar: a cluster randomized controlled trial. BMC Pregnancy and Childbirth. 2014;14(1):29.
39. Nu UT, Pervin J, Rahman AMQ, Rahman M, Rahman A. Determinants of care-seeking practice for neonatal illnesses in rural Bangladesh: A community-based cross-sectional study. PLOS ONE. 2020;15(10):e0240316.
40. Rahman A, Moran A, Pervin J, Rahman A, Rahman M, Yeasmin S, et al. Effectiveness of an integrated approach to reduce perinatal mortality: recent experiences from Matlab, Bangladesh. BMC Public Health. 2011;11(1):914.
41. Ahmed S, Khan MM. Is demand-side financing equity enhancing? Lessons from a maternal health voucher scheme in Bangladesh. Social Science & Medicine. 2011;72(10):1704-10.
42. Kamal N, Curtis S, Hasan MS, Jamil K. Trends in equity in use of maternal health services in urban and rural Bangladesh. International Journal for Equity in Health. 2016;15(1):27.
43. El Arifeen S, Hill K, Ahsan KZ, Jamil K, Nahar Q, Streatfield PK. Maternal mortality in Bangladesh: a Countdown to 2015 country case study. Lancet. 2014;384(9951):1366-74.
44. Huda TM, Chowdhury M, El Arifeen S, Dibley MJ. Individual and community level factors associated with health facility delivery: A cross sectional multilevel analysis in Bangladesh. PLOS ONE. 2019;14(2):e0211113.
45. Dhakal PM, Shrestha MM, Baral DM, Pathak SM. Factors Affecting the Place of Delivery among Mothers Residing in Jhorahat VDC, Morang, Nepal. Int J Community Based Nurs Midwifery. 2018;6(1):2-11.
46. Benova L, Tunçalp Ö, Moran AC, Campbell OMR. Not just a number: examining coverage and content of antenatal care in low-income and middle-income countries. BMJ Global Health. 2018;3(2):e000779.
47. Gebresilassie B, Belete T, Tilahun W, Berhane B, Gebresilassie S. Timing of first antenatal care attendance and associated factors among pregnant women in public health institutions of Axum town, Tigray, Ethiopia, 2017: a mixed design study. BMC Pregnancy and Childbirth. 2019;19(1):340.
48. Okedo-Alex IN, Akamike IC, Ezeanosike OB, Uneke CJ. Determinants of antenatal care utilisation in sub-Saharan Africa: a systematic review. BMJ Open. 2019;9(10):e031890.
49. Pulok MH, Sabah MN-U, Uddin J, Enemark U. Progress in the utilization of antenatal and delivery care services in Bangladesh: where does the equity gap lie? BMC Pregnancy and Childbirth. 2016;16(1):200.
50. Yaya S, Idriss-Wheeler D, Shibre G, Amouzou A, Bishwajit G. Prevalence of institutional delivery and its correlates amongst women of reproductive age in Mozambique: a cross-sectional analysis. Reproductive Health. 2020;17(1):49.
51. Sarker BK, Rahman M, Rahman T, Rahman T, Rahman F, Khalil JJ, et al. Factors associated with calendar literacy and last menstrual period (LMP) recall: a prospective programmatic implication to maternal health in Bangladesh. BMJ Open. 2020;10(12):e036994.
52. Gulliford M, Naithani S, Morgan M. What is 'continuity of care'? Journal of health services research & policy. 2006;11:248-50.

53. Hodnett E. Continuity of caregivers for care during pregnancy and childbirth (Cochrane Review). Cochrane database of systematic reviews (Online). 2000;1:CD000062.

Supporting Information

S1 Appendix. Associations of socio-economic characteristics and outcome variables -unadjusted odds ratios.

S2 Appendix. Associations of Skilled Birth attendance at delivery with socio-economic determinants.

S1 Dataset.

S1 Questionnaire.

Figure

Click here to access/download;Figure;Fig 1.tif

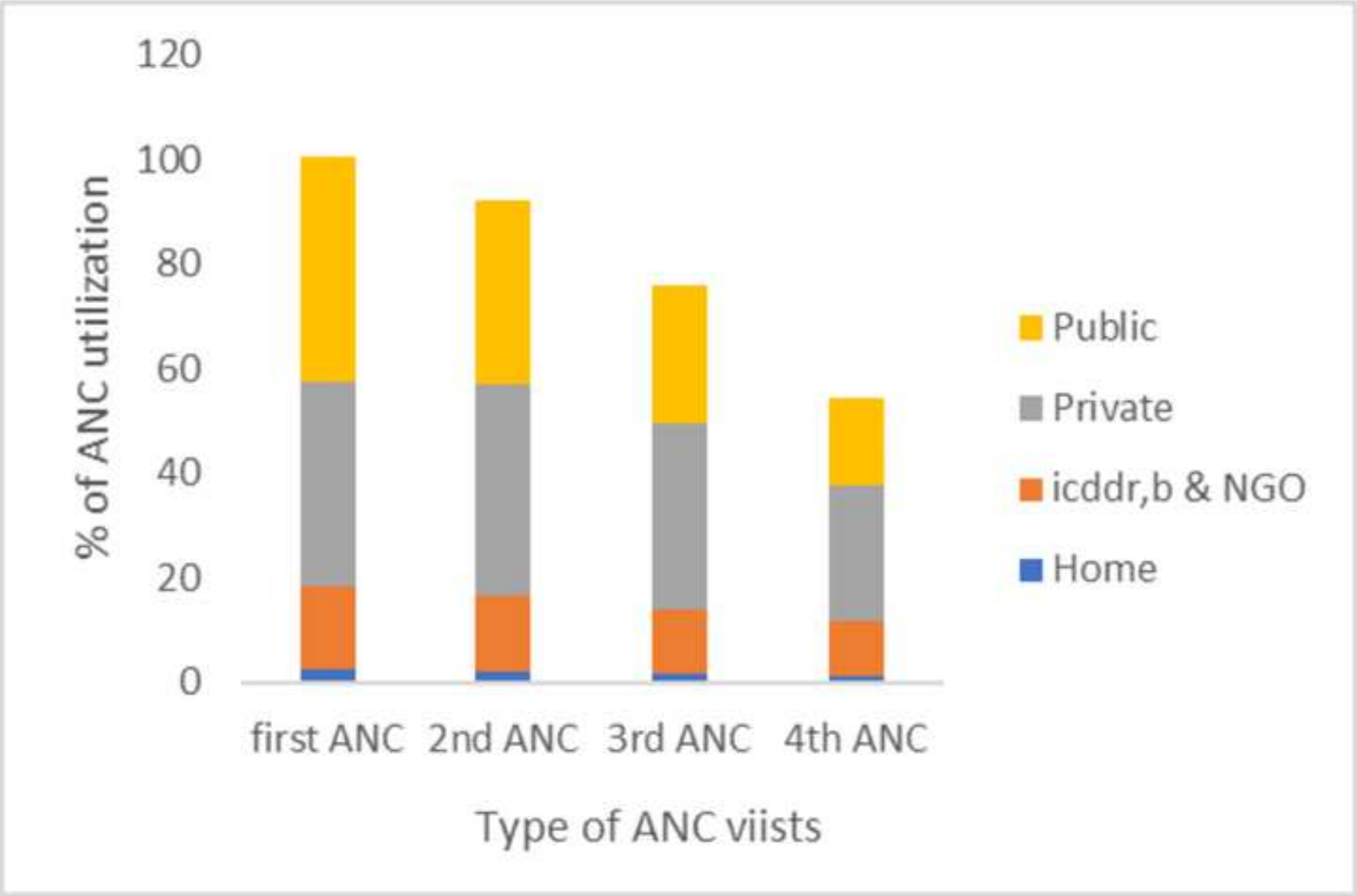

Figure

Click here to access/download;Figure;Fig 2.tif

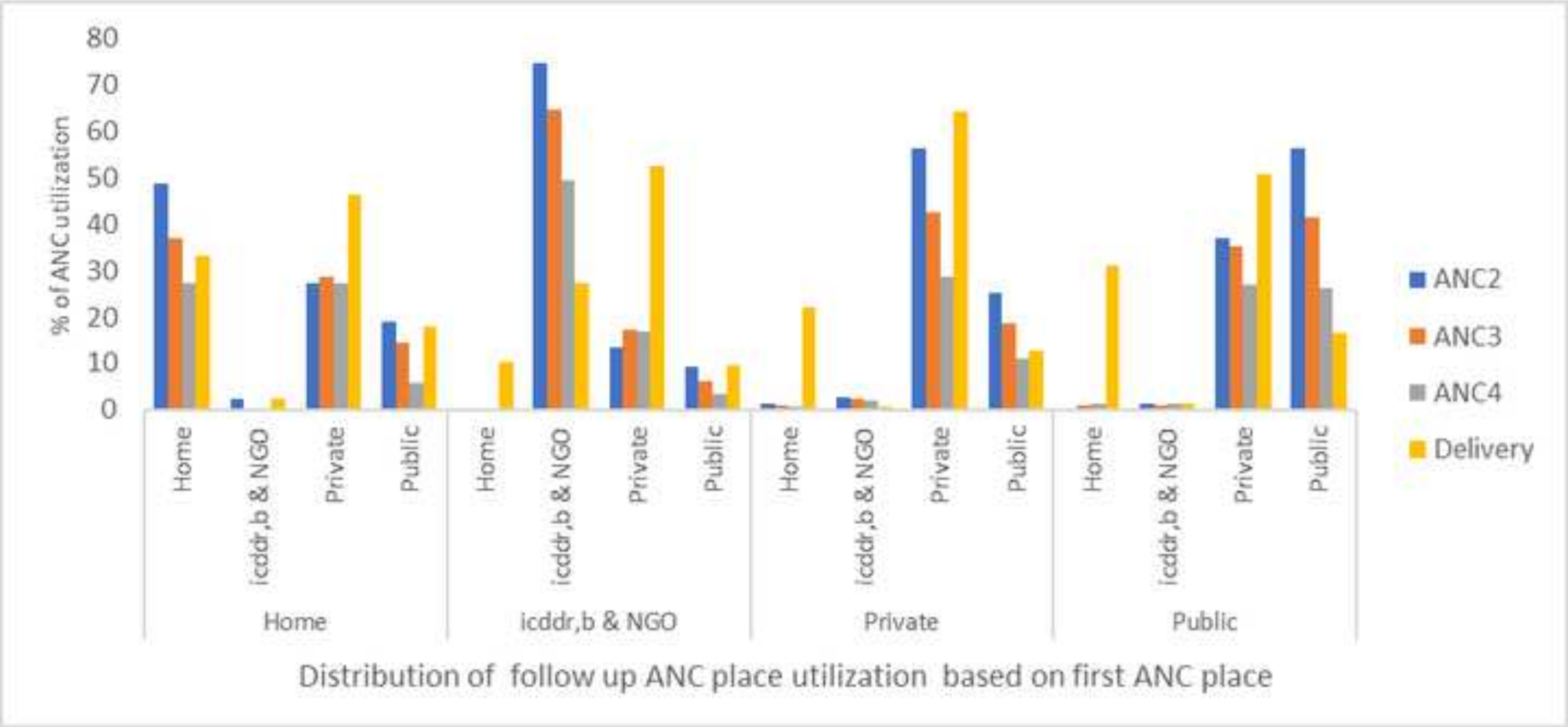

Figure

Click here to access/download;Figure;Fig 3.tif

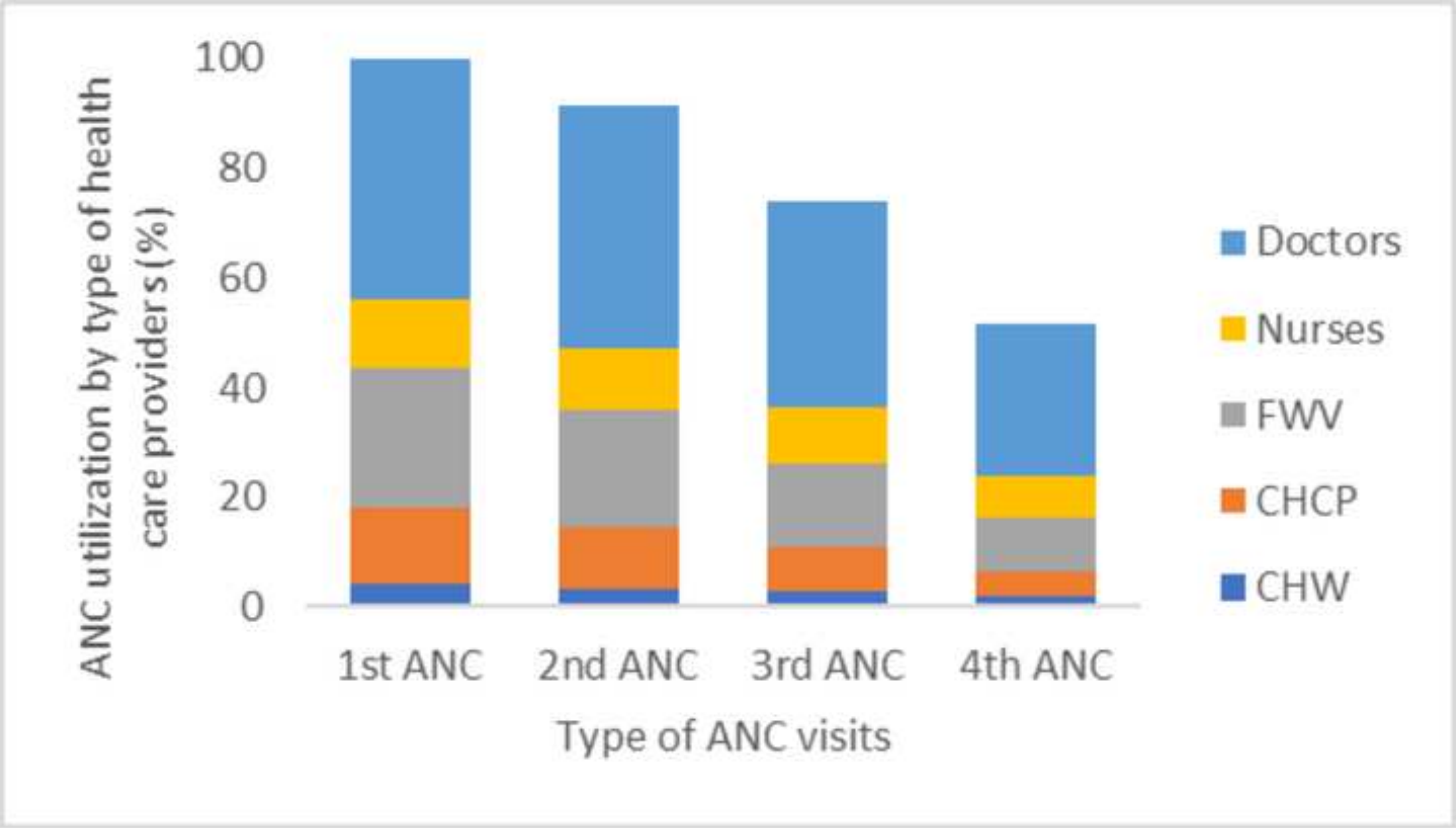

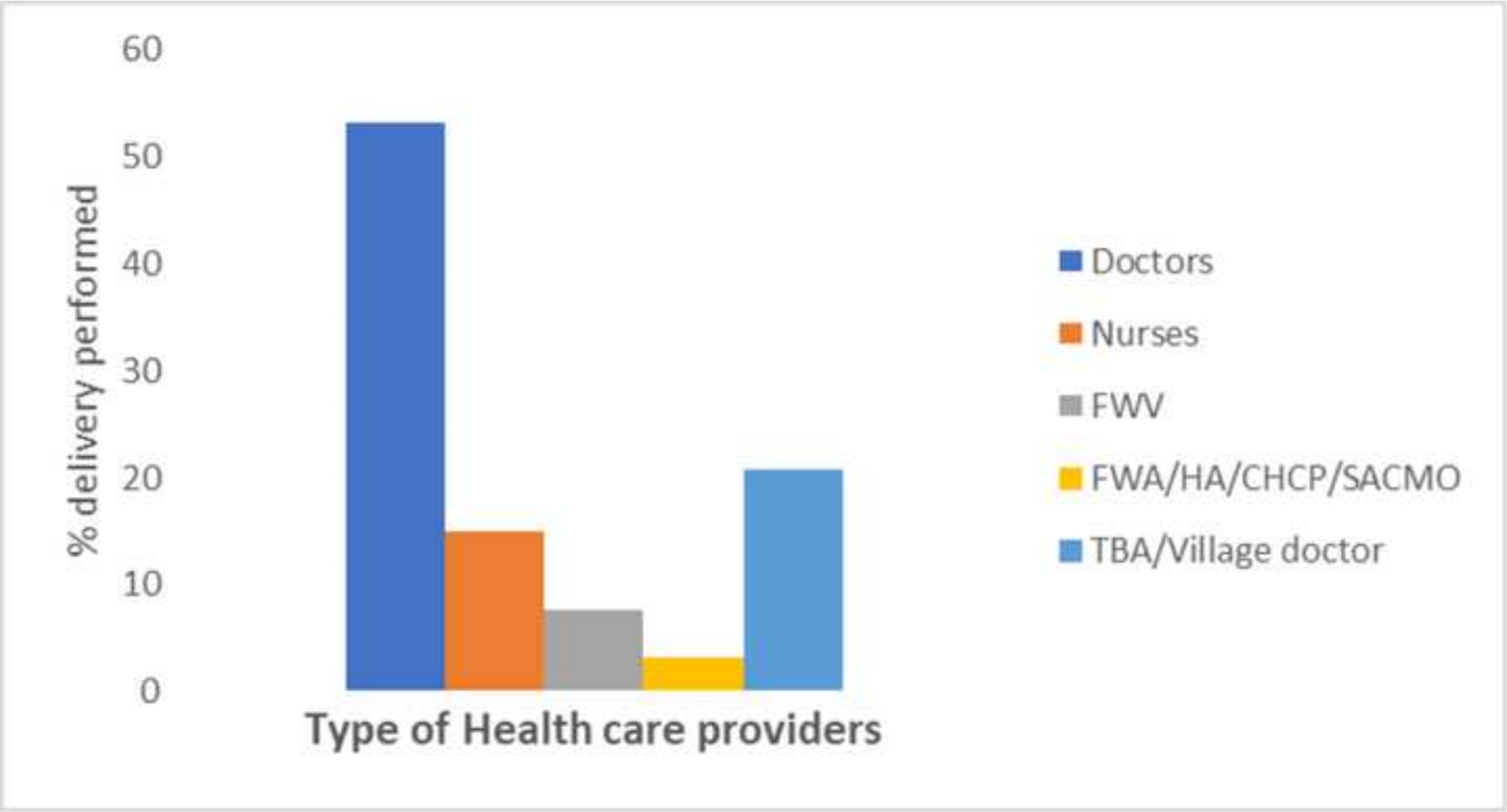
60
50
40
30
20
10
0
% delivery performed
Type of Health care providers
Doctors
Nurses
FWV
FWA/HA/CHCP/SACMO
TBA/Village doctor

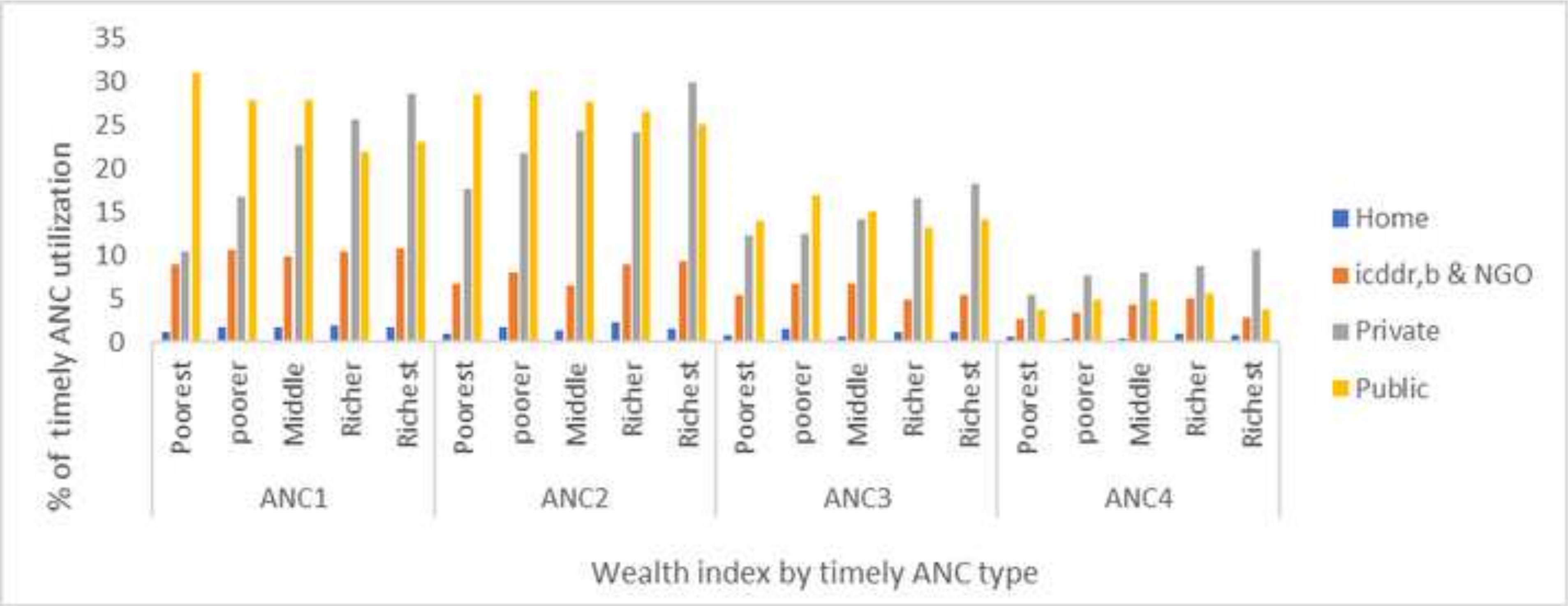

35
30
25
20
15
10
5
0
% of timely ANC utilization
Poorest
poorer
Middle
Richer
Richest
ANC1
ANC2
ANC3
ANC4
Home
icddr,b & NGO
Private
Public
Wealth index by timely ANC type

Figure

Click here to access/download;Figure;Fig 6 .tif

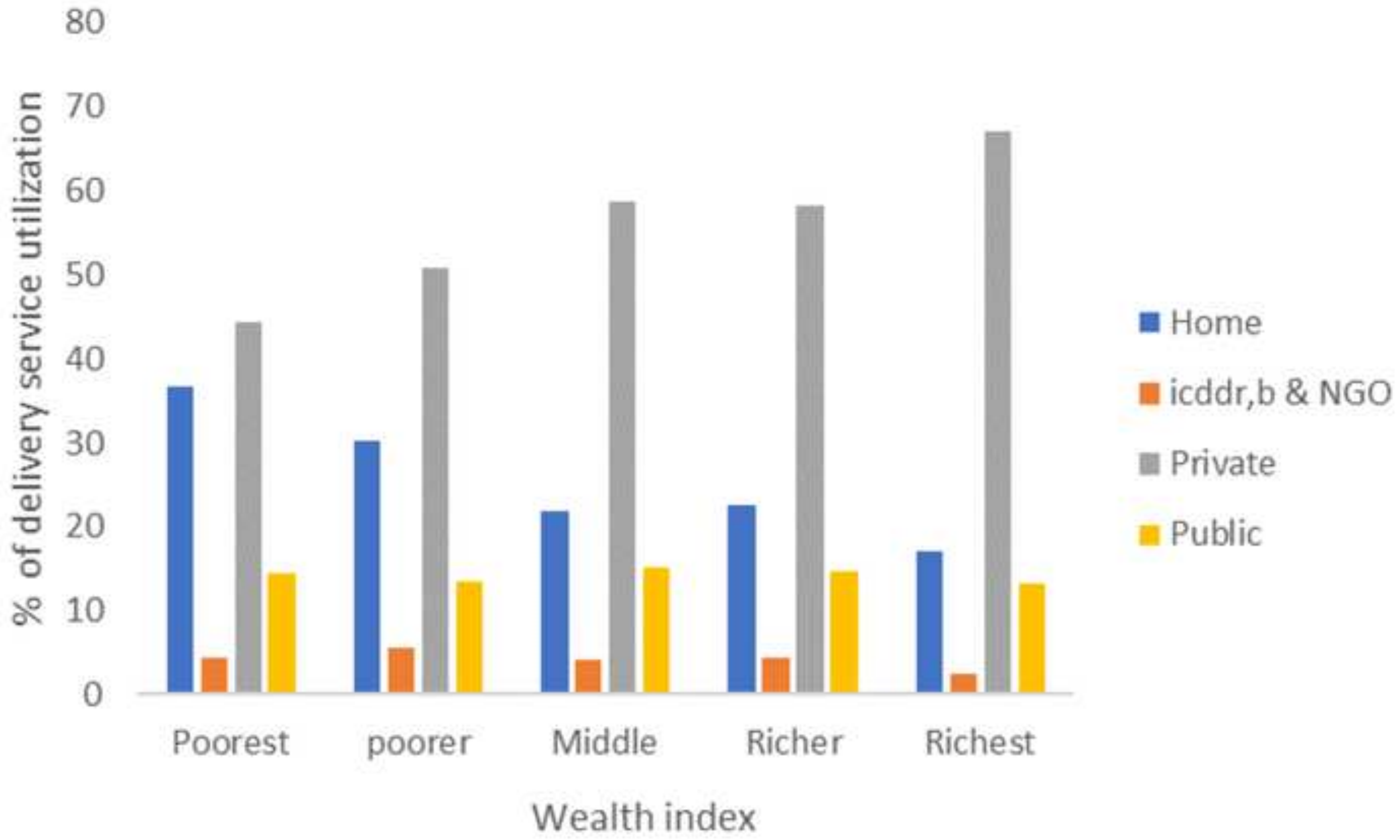

Click here to access/download
Supporting Information
S1 Appendix .docx

Click here to access/download
Supporting Information
S2 Appendix .docx

Click here to access/download
Supporting Information
S1 dataset.dta

Click here to access/download
Supporting Information
S1 Questionnaire.pdf
